# Catechol-linker and receptor-mediated site-specific delivery of bortezomib against non-small cell lung cancer

**DOI:** 10.1016/j.jbc.2025.111095

**Published:** 2025-12-22

**Authors:** Mohan Indhu, Valappil Sisila, Devandran Jayasurya, Niraikulam Ayyadurai

**Affiliations:** 1 Department of Biochemistry and Biotechnology, Council of Scientific and Industrial Research (CSIR) - Central Leather Research Institute, Chennai, Tamil Nadu, India; 2 Academy of Scientific and Innovative Research (AcSIR), Ghaziabad, India; 3 Department of Pharmacology, The Tamil Nadu Dr M.G.R. Medical University, Chennai, Tamil Nadu, India

**Keywords:** receptor-binding domain, 3,4-dihydroxyphenyl-L-alanine, bortezomib, catechol-boronate ester, bio-orthogonal conjugation, angiotensin-converting enzyme-2, site-specific delivery

## Abstract

Angiotensin-converting enzyme-2 (ACE2) receptor-targeting bio-orthogonally conjugated bortezomib (BTZ) was site-specifically delivered against non-small cell lung cancer (NSCLC). Through rational screening, three ACE2 receptor-binding domain (RBD) variants (mutant RBD1, mutant RBD2, and mutant RBD3) were identified to introduce the genetic linker DOPA (3,4-dihydroxyphenyl-L-alanine) to bio-orthogonally load BTZ through a catechol-boronate ester with enhanced receptor binding. Extensive biophysical characterization, such as UV-Vis spectroscopy, B^11^-NMR spectroscopy, RP-HPLC, microscale thermophoresis, and X-ray photoelectron spectroscopy, confirms the homogeneous preparation and controlled release of BTZ. *In vitro* analysis, including 2D monolayer and 3D spheroid models, revealed the site-specific and controlled release of BTZ through ACE2 receptor-mediated endocytosis in subcellular endosomes, which inhibits the proteasome function of NSCLC cells (A549). Finally, *in vivo,* animal studies (Lewis lung carcinoma (LLC1) cells-induced C57BL/6 mice) showed significant inhibition of lung tumor growth with reduced toxicity in normal ACE2-expressing organs. To our knowledge, site-specific labeling and high-potential bio-orthogonal delivery of BTZ in the NSCLC were demonstrated for the first time. More attractively, this concept strengthens future applications for delivering other boronic acid-containing therapeutics against metabolic and cardiovascular diseases.

Non-small cell lung cancer (NSCLC) is the most prevalent subtype of lung cancer, accounting for more than 85% of cases globally and is frequently linked to a poor prognosis (1). The FDA-approved bortezomib (BTZ) is a member of a class of drugs called proteasome inhibitors. This boronic acid-based dipeptide shows potential in NSCLC treatment by reversibly binding to the catalytic site of the 26S proteasome in malignant cells (2, 3). Despite its efficacy, BTZ faces limitations due to its poor stability, low bioavailability, inefficient penetration into solid tumors, interactions with certain natural compounds, and considerable harm to healthy tissues (4, 5). Targeted drug delivery strategies have proven highly effective in addressing these challenges and improving therapeutic outcomes for NSCLC (6). The emergence of the coronavirus disease 2019 (COVID-19) pandemic reveals that patients with lung cancer are more susceptible to infection than healthy individuals (7). An angiotensin-converting enzyme 2 (ACE2), the receptor for severe acute respiratory syndrome coronavirus 2 (SARS-CoV-2), has been reported to be upregulated in lung tumors (8, 9). However, ACE2 expression shows considerable heterogeneity depending on NSCLC tumor subtype, tumor microenvironment, and disease stage (10, 11). Several bioinformatics analyses further demonstrated that ACE2 expression is elevated in both lung adenocarcinoma (LUAD) and lung squamous cell carcinoma (LUSC) subtypes of NSCLC compared to normal lung tissues (12), with particularly high levels in LUAD (13). These findings motivated interest in exploring ACE2 as a potential therapeutic target in NSCLC. The most common targeted drug delivery strategies exploit features of the tumor microenvironment, such as acidity, or target overexpressed receptors on cancer cells to improve specificity (14, 15). The carriers based on receptors, including monoclonal antibodies, small-molecule inhibitors, or ligands for receptors like human epidermal growth factor receptor 2, epidermal growth factor receptor, folate, or transferrin, have demonstrated therapeutic potential (16, 17, 18, 19). However, the heterogeneity of these carriers frequently affects their pharmacokinetics, stability, and efficacy (20, 21, 22, 23, 24). It is paramount to identify innovative carrier platforms that could enable efficient drug delivery and biocompatibility. The key requirements are maintaining their structural or functional integrity and allowing homogenous drug loading.

Herein, the strategy for directing BTZ to NSCLC cells hinges on the specific interaction between the cell surface receptor ACE2 and the SARS-CoV-2 spike receptor-binding domain (RBD) (25). As the native RBD (amino acids Arg319-Phe541) is primarily responsible for ACE2 binding, we aimed to develop a novel RBD protein-based targeting moiety. We rationally designed three different RBD variants (mutant RBD1, mutant RBD2, and mutant RBD3) to introduce a genetic catechol linker 3,4-dihydroxyphenyl-L-alanine (DOPA) for bio-orthogonal conjugation of BTZ through dynamic covalent boronate chemistry. This enables the formation of boronate ester that is stable at physiological pH, maintaining BTZ in its inactive form, while dissociating under acidic conditions of tumor microenvironment (extracellular environment, late endosome, or lysosome). Thus, the genetic linker DOPA-encoded protein-drug (RBDdopa-BTZ) conjugate selectively targets ACE2-overexpressing NSCLC cells and releases BTZ through receptor-mediated endocytosis (Fig. 7). This pH-responsive, receptor-mediated cargo has the potential to overcome the drawbacks of BTZ delivery. It improves the antitumor efficacy and activates the proteasome inhibitory action *in vitro* using 2D monolayer and 3D spheroid models. In a syngeneic lung cancer model, the RBDdopa-BTZ conjugate showed potent antitumor efficacy against ACE2-expressing lung tumors with reduced toxicity in normal ACE2-expressing organs (liver, kidney, and heart).

**Figure 1:**
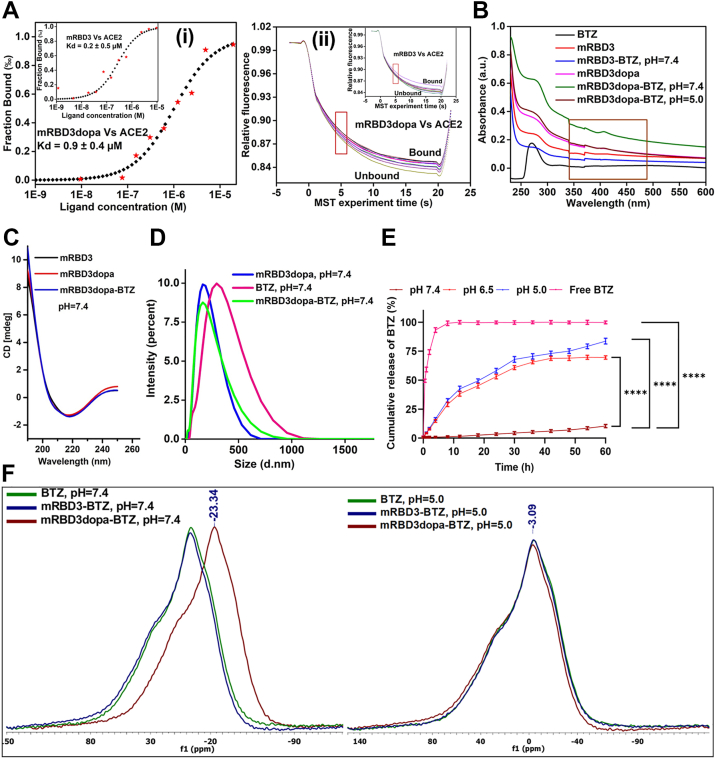
**Site-specific, bio-orthogonal conjugation and pH-responsive BTZ Release.***A*, binding kinetics of mRBD3 and mRBD3dopa with ACE2 using MST (i) The *black square* indicates the curve generated by Monolith binding affinity Nano Temper Analysis 1.2.231 software, and the red dot indicates the fluorescent intensity (fraction bound) on protein-receptor interaction, and (ii) T jump plot of mRBD3 and mRBD3dopa vs. ACE2 shows how the temperature changed when the thermal activation by the IR laser was turned on, and the changes in the fluorescence signal occurred in the MST capillaries and ended within a few seconds are denoted by temperature jumps (T jumps). K_D_ values are reported as mean ± SD using three independent protein preparations, each measured in technical triplicate. The binding curves shown are representative of three independent ligand preparations and labeling reactions. *B*, representative UV-vis spectra of the catechol-boronate complex measured at pH 7.4 and 5.0. *C*, representative CD spectrum of mRBD3, mRBD3dopa and mRBD3dopa-BTZ conjugate at pH 7.4. *D*, homogeneity analysis using DLS. *E*, quantification of cumulative BTZ release profile using UV-Vis spectroscopy (λ = 270 nm) at pH 7.4, 6.5, and 5.0. Free BTZ is tested as control in the absence of drug carrier. Data represent mean ± SD from three independent sample preparations, and each time point was measured in technical triplicate. Error bars denote standard deviation. Statistical analysis was made using a two-way ANOVA with multiple comparisons and Tukey’s *post hoc* correction, ∗∗∗∗*p* < 0.0001 and significance is displayed for the final 60 h time point. *F*, representative B^11^-NMR spectrum of free BTZ and conjugates at pH 7.4 and 5.0.

**Figure 2:**
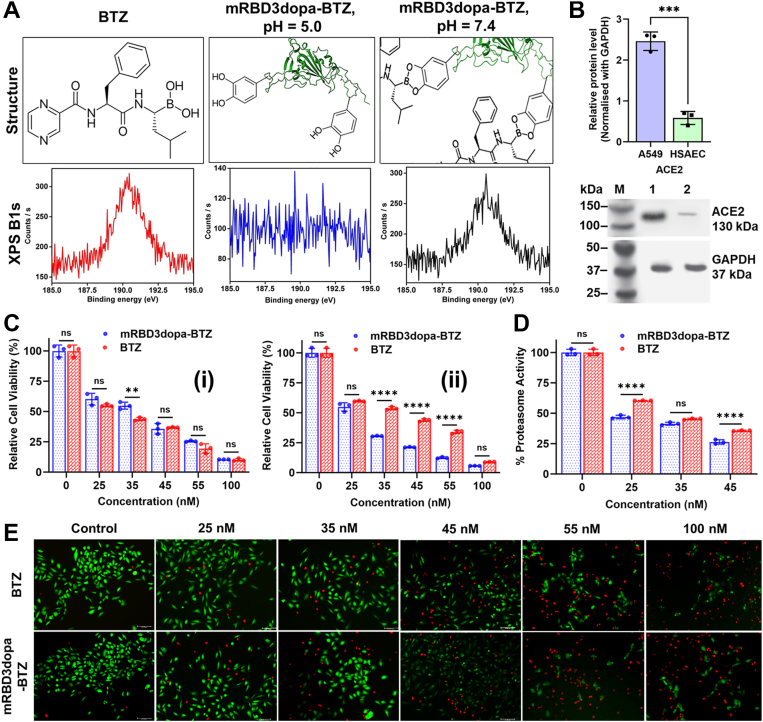
**Functional mechanism of ester-bond stability and evaluation of selective cytotoxicity in ACE2-high A549 cells.***A*, representative XPS spectra showing the surface chemistry of free BTZ and the conjugate at pH 7.4 and 5.0. *B*, Western blot quantification of ACE2 expression (i) Protein expression levels were quantified and normalized to GAPDH, and (ii) Detection of ACE2 protein expression in A549 cells compared with HSAEC cells, Lane M: Marker, Lane 1: A549 cells and Lane 2: HSAEC cells. GAPDH was used as an internal control of protein loading. Band intensity was calculated by ImageJ and plotted using GraphPad Prism 10.1.0. Data are presented as mean ± SD from at least three independent biological replicates, each done in three technical replicates. The data points representing the mean of each independent biological replicate are overlaid. Error bars denote standard deviation across the biological replicates. Statistical analysis was made using an unpaired two-tailed *t* test, ∗∗∗*p* < 0.001. *C*, dose-dependent cytotoxicity of mRBD3dopa-BTZ and free BTZ in ACE2-high A549 cells, assessed by MTT assay at (i) 24 h, and (ii) 48 h. Data are presented as mean ± SD from at least three independent biological replicates, each done in five technical replicates. The data points representing the mean of each independent biological replicate are overlaid. Error bars denote standard deviation across the biological replicates. Statistical analysis was made using a using two-way ANOVA with multiple comparisons and Tukey’s *post hoc* correction, ∗∗*p* < 0.01, ∗∗∗∗*p* < 0.0001, ns = not significant. *D*, proteasome activity in A549 cells cultured as 2D monolayers for 48 h. Data are presented as mean ± SD from at least three independent biological replicates, each done in five technical replicates. The data points representing the mean of each independent biological replicate are overlaid. Error bars denote standard deviation across the biological replicates. Statistical analysis was made using a using two-way ANOVA (Factors: treatment × concentration) with multiple comparisons and Tukey’s *post hoc* correction, ∗∗∗∗*p* < 0.0001, ns = not significant. *E*, live/dead cell analysis using FDA/PI staining after 48 h of incubation. The scale bar represents 100 μm.

**Figure 3:**
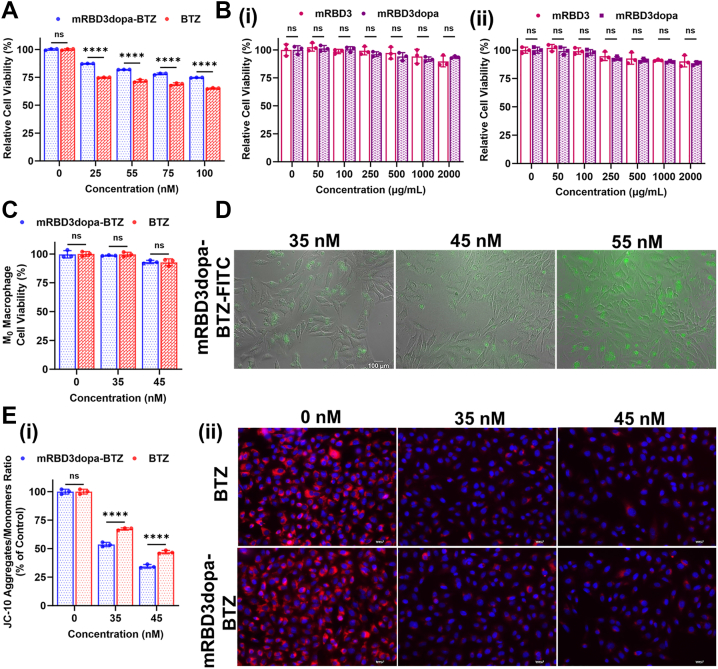
**Safety, specificity and assessment of apoptosis.***A*, cytotoxicity of mRBD3dopa-BTZ and free BTZ in ACE2-low HSAEC cells after 48 h treatment. Data are presented as mean ± SD from at least three independent biological replicates, each done in four technical replicates. The data points representing the mean of each independent biological replicate are overlaid. Error bars denote standard deviation across the biological replicates. Statistical analysis was made using a using two-way ANOVA with multiple comparisons and Tukey’s *post hoc* correction, ∗∗∗∗*p* < 0.0001, ns = not significant. *B*, cytotoxicity assessment of mRBD3 and mRBD3dopa in A549 cells after (i) 24 h and (ii) 48 h treatments. Data are presented as mean ± SD from at least three independent biological replicates, each done in four technical replicates. The data points representing the mean of each independent biological replicate are overlaid. Error bars denote standard deviation across the biological replicates. Statistical analysis was made using a using one-way ANOVA with multiple comparisons and Tukey’s *post hoc* correction, ns = not significant compared to control. *C*, cytocompatibility evaluation of mRBD3dopa-BTZ and free BTZ in THP-1 derived macrophages (M0 phenotype). Data are presented as mean ± SD from at least three independent biological replicates, each done in six technical replicates. The data points representing the mean of each independent biological replicate are overlaid. Error bars denote standard deviation across the biological replicates. Statistical analysis was made using a using two-way ANOVA with multiple comparisons and Tukey’s *post hoc* correction, ns = not significant. *D*, cellular uptake in A549 cells was determined by fluorescence microscopy for 6 h. The scale bar represents 100 μm. E, Induction of mitochondrial intrinsic apoptosis (i) Effect of mRBD3dopa-BTZ and free BTZ on MMP in A549 cells using the JC-10 dye. Data are presented as mean ± SD from at least three independent biological replicates, each done in five technical replicates. The data points representing the mean of each independent biological replicate are overlaid. Error bars denote standard deviation across the biological replicates. Statistical analysis was made using a using two-way ANOVA (Factors: treatment × concentration) with multiple comparisons and Tukey’s *post hoc* correction, ∗∗∗∗*p* < 0.0001, ns = not significant. (ii) Detection of MMP using rhodamine 123 staining. The scale bar represents 100 μm.

**Figure 4:**
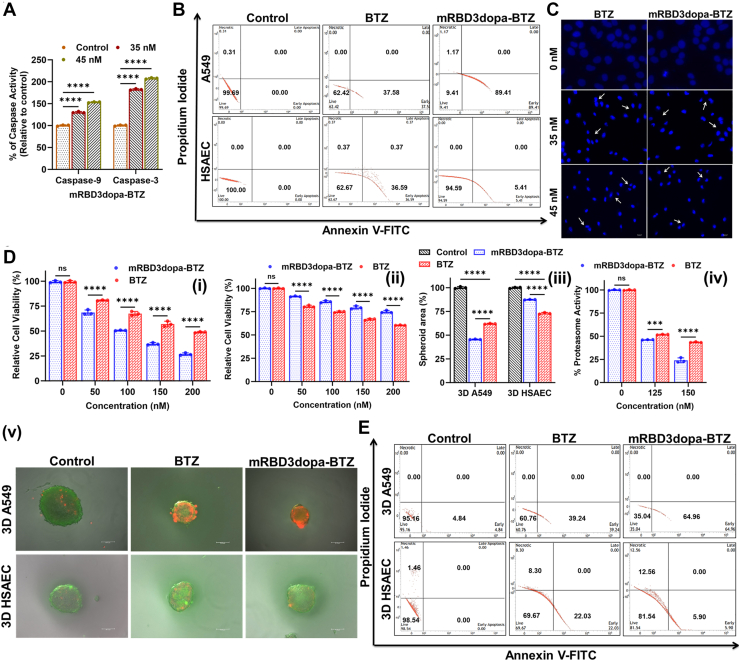
**Apoptotic mechanism of the ACE2-targeted mRBD3dopa-BTZ in 2D monolayer and 3D spheroid models.***A*, evaluation of caspase-3 and caspase-9 activities in A549 cells treated with mRBD3dopa-BTZ. Data are presented as mean ± SD from at least three independent biological replicates, each done in five technical replicates. The data points representing the mean of each independent biological replicate are overlaid. Error bars denote standard deviation across the biological replicates. Statistical analysis was made using a using two-way ANOVA (Factors: caspase type × concentration) followed by Tukey’s multiple comparisons test to compare each treatment group with the corresponding control within each caspase, ∗∗∗∗*p* < 0.0001. *B*, analysis of cell apoptosis and death in A549 and HSAEC Cells (2D monolayer) by flow cytometry. The dot plots shown are representative of three independent biological experiments. *C*, Nuclear condensation and fragmentation analysed by Hoechst-33342 staining. The scale bar represents 100 μm. *D*, cytotoxicity of mRBD3dopa-BTZ and free BTZ in 3D spheroid cultures after 48 h treatment (i) 3D A549, (ii) 3D HSAEC spheroids. Data are presented as mean ± SD from at least three independent biological replicates, each done in five technical replicates. The data points representing the mean of each independent biological replicate are overlaid. Error bars denote standard deviation across the biological replicates. Statistical analysis was made using a using two-way ANOVA with multiple comparisons and Tukey’s *post hoc* correction, ∗∗∗∗*p* < 0.0001, ns = not significant. (iii) Effect of mRBD3dopa-BTZ and free BTZ on 3D spheroid area of A549 and HSAEC cultures. Spheroid areas were quantified using ImageJ and normalized to untreated controls. Data are presented as mean ± SD from at least three independent biological replicates, each done in six technical replicates. The data points representing the mean of each independent biological replicate are overlaid. Error bars denote standard deviation across the biological replicates. Statistical analysis was made using a two-way ANOVA with multiple comparisons and Tukey’s *post hoc* correction, ∗∗∗∗*p* < 0.0001. (iv) Evaluation of proteasome activity in the 3D A549 spheroid model. Data are presented as mean ± SD from at least three independent biological replicates, each done in five technical replicates. The data points representing the mean of each independent biological replicate are overlaid. Error bars denote standard deviation across the biological replicates. Statistical analysis was made using a two-way ANOVA (Factors: treatment × concentration) with Tukey’s multiple comparisons, ∗∗∗*p* < 0.001, ∗∗∗∗*p* < 0.0001, ns = not significant. (v) Live/dead cell analysis of 3D A549 and 3D HSAEC spheroids using FDA/PI staining with incubation time 48 h. The scale bar represents 100 μm. *E*, analysis of cell apoptosis and death in A549 and HSAEC Cells (3D spheroids) by flow cytometry. The dot plots shown are representative of three independent biological experiments.

**Figure 5:**
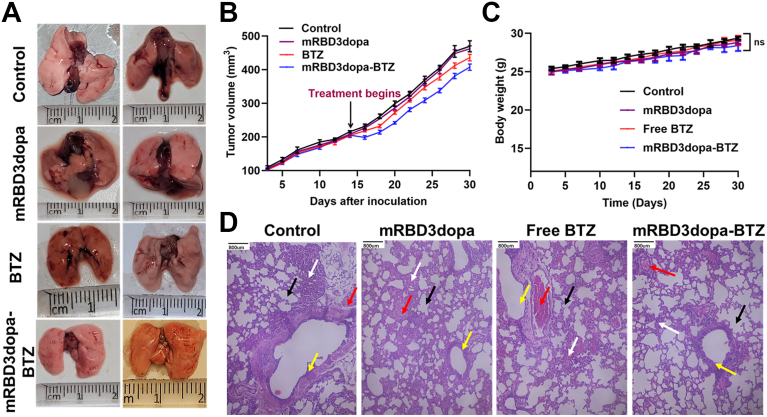
***In vivo* antitumor efficacy of mRBD3dopa-BTZ in a LLC1 tumor induced C57BL/6 mice.***A*, representative photographs of excised lungs collected from LLC1 tumor-bearing mice following the indicated treatments after a 30-days observation period (biological replicates n = 6 mice per group). *B,* assessment of tumor growth inhibition in a syngeneic lung cancer model. Tumor growth was presented as the mean tumor volume (mm^3^) ± SEM. Tumor volume was determined by caliper measurements and calculated as length × width^2^/2. Error bars represent standard error of the mean. Statistical analysis was performed using a two-way repeated-measures ANOVA (treatment × time; Geisser–Greenhouse correction) followed by Tukey’s multiple comparison test for group-wise differences at each time point. *C*, body weight (g) of mice monitored over 30 days. Data are presented as mean ± SD (biological replicates n = 6 mice per group). Error bars represent standard deviation. Statistical analysis was made using a two-way ANOVA with multiple comparisons and Tukey’s *post hoc* correction, ns = not significant. *D*, representative H&E-stained sections of mice lung tissue after treatments: dysplastic epithelium (*yellow arrow*), alveoli (b*lack arrow*) surrounded by inflammatory cells composed mainly of plasma cells and lymphocytes (*white arrow*), and normal blood vessels (*red arrow*). The scale bar represents 800 μm.

**Figure 6:**
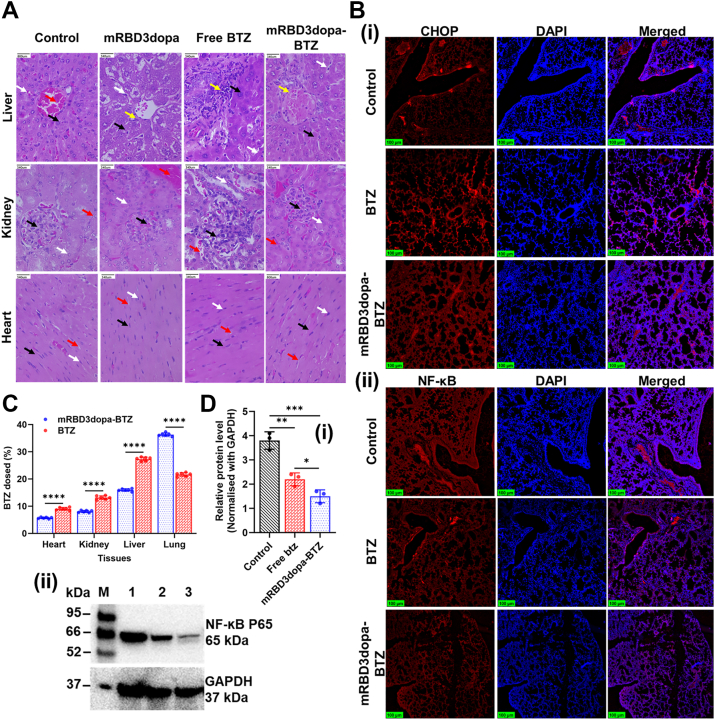
**Organ-protective efficacy, selectivity, and apoptotic induction of mRBD3dopa-BTZ *in vivo*.***A*, representative H&E-stained sections of mice organs collected after different treatments: in the liver-hepatocytes (*black arrow*), central vein (*red arrow*), portal triad (*yellow arrow*), and sinusoidal spaces (*white arrow*); in the kidney-glomeruli (*black arrow*), tubules (*white arrow*), and the interstitium (*red arrow*); and in the heart-cardiomyocytes (*black arrow*), myonuclei (*white arrow*), and intercalated disc space (*red arrow*). The scale bar represents 340 μm. *B*, representative immunohistochemical staining showing the expression of (i) CHOP, and (ii) NF-kB. The scale bar represents 100 μm. *C*, biodistribution of BTZ in major organs of tumor-bearing mice 12 h post-injection was quantified by ICP-MS and expressed as the percentage of total injected boron. Data represent the mean ± SD (biological replicates n = 6 mice per group), with each sample measured in technical triplicate. The data points representing the mean of each independent biological replicate are overlaid. Error bars denote standard deviation across the biological replicates. Statistical analysis was made using a two-way ANOVA with multiple comparisons and Tukey’s *post hoc* correction, ∗∗∗∗*p* < 0.0001. *D*, Western blots quantification of NF-κB expression following treatment (i) Protein expression levels were quantified and normalized to GAPDH, and (ii) Representative Western blot showing NF-κB inhibition, Lane M: Marker, Lane 1: Control, Lane 2: Free BTZ and Lane 3: mRBD3dopa-BTZ. Band intensity was calculated by ImageJ and plotted using GraphPad Prism 10.1.0. Data represent the mean ± SD from three independent biological replicates (n = 3 mice per group). Error bars denote standard deviation across the biological replicates. Statistical analysis was performed using a one-way ANOVA with multiple comparisons and Tukey’s *post hoc* correction, ∗*p* < 0.05, ∗∗*p* < 0.01, ∗∗∗*p* < 0.001.

**Figure 7:**
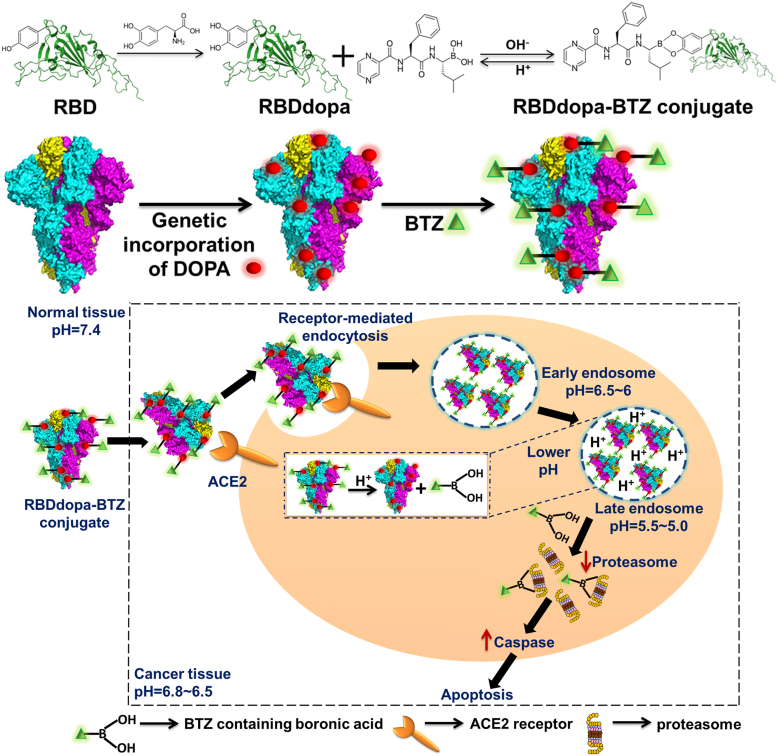
**Site-specific delivery of bio-orthogonally conjugated bortezomib against non-small cell lung cancer cells**.

## Results

### Rational design of SARS-CoV-2 RBD variants

The three SARS-CoV-2 RBD variants (mRBD1, mRBD2, and mRBD3) were rationally designed to improve structural stability, enhance recombinant expression, and enable site-specific DOPA incorporation while preserving ACE2-binding affinity. These RBD variants (mRBD1-3) were derived from the wild-type RBD (nRBD, residues 319–541) without altering the overall protein conformation. In mRBD1, the serine residues were conservatively substituted with threonine to reduce protease susceptibility. In mRBD2, potential N-linked glycosylation sites were removed to enhance expression yield. In mRBD3, the structurally unaffected tyrosine residues were substituted with structurally similar aromatic amino acids to facilitate DOPA incorporation while maintaining ACE2 binding. Together, these modifications generated three distinct RBD mutants that retained ACE2 binding affinity comparable to the nRBD. The detailed sequence information of nRBD, mRBD1, mRBD2, and mRBD3, collectively named RBDs, is provided in (Table S1). The structural stability of ACE2, RBDs, and genetic linker DOPA incorporated RBDs (nRBDdopa, mRBD1dopa, mRBD2dopa, and mRBD3dopa), collectively named RBDdopa’s, was predicted using AlphaFold and visualized in PyMOL. Molecular docking studies predicted ACE2 interaction and indicated stable polar contact formation. Based on the docking scores (Fig. S1 and Table S2), the RBDs and RBDdopa’s showed progressive improvement in ACE2-binding affinity compared to the wild-type nRBD. Among them, mRBD3 and DOPA-incorporated mRBD3 achieved the highest docking scores of −866.5 and −873.9 kcal/mol, respectively. These results confirm that the rational design strategy preserved functional ACE2 interaction while enabling bio-orthogonal conjugation for drug delivery.

### Ribosomal synthesis of RBDs and RBDdopa’s by expanding the genetic code

The nucleotide sequences of the RBDs were codon optimized and cloned into the pQE80L plasmid for protein production. The recombinant RBDs expression was optimized in *Escherichia coli* strain JW2581 using 1 mM IPTG. Simultaneously, the genetic linker DOPA was introduced into the RBDs by the selective pressure incorporation (SPI) method after depleting the amino acid tyrosine (26, 27). The expression efficacy of RBDdopa’s compared with RBDs was optimized. The expressed proteins were purified using a nickel-nitrilotriacetic acid (Ni-NTA) affinity column and analyzed by sodium dodecyl sulfate-polyacrylamide gel electrophoresis (SDS-PAGE) (Fig. S2). The expression yield for the RBDs ranged from 11 to 13 mg/L of *E. coli* culture, while the RBDdopa’s yielded between 6 and 8 mg/L following purification. The quantitative substitution of tyrosine by DOPA in RBDs was confirmed by matrix-assisted laser desorption/ionization Time-of-Flight (MALDI-TOF) mass spectrometry. The variants nRBDdopa, mRBD1dopa, and mRBD2dopa each had 15 tyrosine residues replaced by DOPA, resulting in an increased total mass of about ∼240 Da per variant. In contrast, the mRBD3dopa variant showed a total mass increase of approximately 160 Da, corresponding to the replacement of 10 tyrosine residues with DOPA. The experimentally observed molecular weights of all proteins aligned with the expected theoretical values, confirming the successful incorporation of DOPA (Fig. S3*A* and Tables S3 and S4).

### Structural and functional characterization of RBDs and RBDdopa’s for bio-orthogonal coupling

The protein samples (RBDs and RBDdopa’s) were subjected to circular dichroism (CD) analysis to resolve the secondary structure of proteins after DOPA incorporation. Both RBDs and RBDdopa’s showed the β-sheet structure; this proves that even after incorporating the genetic linker DOPA, the overall secondary structure remained unchanged (Fig. S3*B*). Furthermore, to confirm that the genetic linker DOPA was incorporated into the RBDs, a redox staining method using nitroblue tetrazolium (NBT) was employed (Fig. S3*C*), which detects proteins containing o-catechols (28). The recombinant ACE2 was expressed, purified, and characterized to study the binding kinetics of RBDs and RBDdopa’s, as confirmed by SDS-PAGE, CD, and MALDI-TOF analysis (Fig. S4). The binding affinity of the proteins (RBDs and RBDdopa’s) to the ACE2 receptor was measured using microscale thermophoresis (MST) by monitoring the binding of fluorescently tagged ACE2 at various concentrations (29). A binding curve was generated by plotting normalized fluorescence against the ligand (ACE2) concentration. Both RBDs and RBDdopa’s showed comparable affinity toward ACE2, yielding close apparent K_D_ values (Fig. S5 and Table S5). Specifically, the titration of mRBD3 and mRBD3dopa yielded K_D_ values of 0.2 ± 0.5 and 0.9 ± 0.4 μM respectively, revealing the stronger binding affinity with the ACE2 receptor among all RBD variants (Fig. 1*A*).

### Site-specific, bio-orthogonal conjugation efficiency and characterization

DOPA catechol chemistry holds significant a potential for drug delivery, as it enables the formation of reversible boronate ester bond at physiological pH, which dissociate into boronic acid and catechol under acidic conditions (30, 31, 32, 33, 34). The purified mRBD3 (1 mg/ml, without genetic linker DOPA) and mRBD3dopa (1 mg/ml, containing 10 DOPA residues) were separately bio-orthogonally conjugated with BTZ in 1× PBS (pH 7.4) at a 10:1 M ratio of BTZ: protein at RT under stirring for 12 h. After conjugation, free BTZ was separated using a 10-kDa cut-off filter through centrifugation at 4000 rcf for 10 min and repeated thrice, resulting in mRBD3-BTZ and mRBD3dopa-BTZ conjugate. The conjugation efficiency was confirmed using MALDI-TOF. The mRBD3-BTZ showed no mass shift and retained its original mass of 24,357.5 Da, inability of BTZ to bind without the genetic linker DOPA. In contrast, the total mass of the mRBD3dopa-BTZ conjugate shifted to 28,019.8 Da, confirming a conjugation efficiency of more than 90% (Fig. S6*A* and Table S6). In ultraviolet-visible (UV-Vis) spectroscopy analysis, all the proteins and their conjugates exhibit absorbance maxima at 280 nm, and free BTZ is established through its distinctive wavelengths at 267 nm. The conjugate, mRBD3dopa-BTZ at pH 7.4, shows a characteristic peak at 408 nm, indicating boron-catechol bonds. Lowering the pH of the solution from 7.4 to 5.0 resulted in decreased peak intensity, suggesting the release of BTZ from the mRBD3dopa, as evidenced by spectra similar to those of mRBD3dopa. In contrast, mRBD3-BTZ at pH 7.4 lacks a catechol group, resulting in the absence of a peak at 408 nm (Fig. 1*B*). The CD spectrum and MST profiles of mRBD3dopa-BTZ revealed a β-sheet structure and a K_D_ value of 1.0 ± 0.4 μM, similar to mRBD3dopa (0.9 ± 0.4 μM). This indicates that the biorthogonal conjugation of BTZ did not significantly alter the secondary structure and conjugate's binding efficiency to the ACE2 receptor, a critical step in targeting cancer cells (Fig. 1*C*). A single intensity peak in a dynamic light scattering (DLS) analysis of a conjugate suggests the absence of protein aggregation or degradation under conjugation conditions, ensuring conjugate homogeneity (Fig. 1*D*).

### Functional mechanism of ester-bond stability and pH-responsive BTZ release from genetically encoded mRBD3dopa

The cleavage kinetics of the boronate ester using mRBD3dopa-BTZ conjugate was performed in PBS solutions at pH 7.4, 6.5, and 5.0, which correspond to the physiological pH in normal tissue and blood, the extracellular environment of tumors, and the subcellular endosome, respectively (35, 36). The weight ratio of cumulative BTZ released to the total BTZ conjugated in the mRBD3dopa was measured by UV/vis spectroscopy at 267 nm as a function of release time (37). Under acidic conditions similar to the endosome (pH 5.0), ∼83.7 ± 2.5% of the BTZ was released from mRBD3dopa over a 60-h period. At an acidic tumor-mimicking environment (pH 6.5), the release was reaching ∼69.5 ± 1.5%, whereas at physiological pH 7.4, release remained minimal at only ∼10.4 ± 1.4%. In contrast, free BTZ diffused rapidly across the dialysis membrane, reaching ∼99.8 ± 1.3% release within 12 h and remaining constant thereafter (Fig. 1*E*). Further, quantification of released BTZ over time (0–60 h) at different pH values (7.4, 6.5, and 5.0) was performed using reversed-phase high-performance liquid chromatography (RP-HPLC). The results showed that only 7.50 ± 2.60% of BTZ were released frothe mRBD3dopa-conjugateat pH 7.4 after 48 h. In contrast, under mildly acidic conditions of the tumor extracellular microenvironment and endosomal compartments (pH 6.5 and 5.0), the release rate was significantly higher, with 69.87 ± 3.10% and 75.90 ± 3.01% of the loaded drug released within the same period. HPLC chromatograms of released BTZ exhibited monodispersed peaks with retention time at 7.25 min. However, the intensity of these characteristic peaks (retention time: 7.25 min) increased gradually along with the extended incubation time (24, 48 and 60 h) under acidic conditions (pH 6.5 and 5.0). These results coincided with quantified UV data and further validating the efficient, acid-triggered cleavage of the catechol-boronate bond (Fig. S6, *B* and *C*). In the boron-11 nuclear magnetic resonance (B^11^-NMR) spectroscopy (26), the free BTZ peak was observed at −3 ppm, whereas the mRBD3dopa-BTZ conjugate revealed a distinct peak at −23 ppm, indicating the formation of a DOPA-boronate complex at pH 7.4. Importantly, the absence of a peak at −3 ppm in the conjugate also confirmed that all the DOPA residues were converted into o-quinone, exhibiting a high propensity for conjugation with BTZ. When the pH was decreased to 5.0, the conjugate displayed a peak at −3 ppm, corresponding to the BTZ peak, indicating that BTZ was released with intact boronic acids under acidic conditions, ensuring its proteasome binding activity. In contrast, the mRBD3-BTZ spectra at both pH values (7.4 and 5.0) showed a peak identical to that of BTZ (−3 ppm), indicating the presence of uncomplexed DOPA-boronate formation (Fig. 1*F*). This confirms the pH-dependent BTZ dissociation from the mRBD3dopa cargo, which could render BTZ non-cell permeable and inactive in normal tissues but allow the BTZ activity to be delivered in acidic cancer microenvironment. Further, X-ray photon spectrum (XPS) (38), indicates the presence of elemental boron spectra at ∼192.33 eV in both boron-containing BTZ and mRBD3dopa-BTZ conjugate at pH 7.4. No boron peaks were observed at pH 5.0, confirming the successful release of BTZ under acidic conditions. This observation validates the pH-dependent release of BTZ from the conjugate (Fig. 2*A*).

### ACE2-mediated targeting and selective cytotoxicity of mRBD3dopa-BTZ in ACE2-overexpressing NSCLC cells

To support our rationale for targeting ACE2, the ACE2 gene expression was analyzed in LUAD and LUSC, the two major subtypes of NSCLC, using the GEPIA2 web server, which integrates data from the TCGA and GTEx datasets. The TCGA datasets revealed that ACE2 expression was significantly higher in both LUAD (n = 483) and LUSC (n = 486) tumor tissues than in normal lung tissues (n = 347 and n = 338, respectively). The GEPIA2 box-plot analysis showed that the median ACE2 transcript levels were slightly higher in LUAD tumors compared with LUSC tumors, and both subtypes exhibited significantly higher expression than normal lung tissue (*p* < 0.05 for both LUAD and LUSC) (Fig. S6*D*). Additionally, the differential expression of ACE2 protein between ACE2-overexpressing human lung carcinoma epithelial cells (A549) and ACE2-low expressing normal human primary small airway epithelial cells (HSAEC) was confirmed by Western blot analysis, with a band detected at ∼130 kDa. As shown in Figure 2*B*, A549 cells exhibited significantly higher ACE2 protein levels (∼4.2 fold) compared to normal HSAEC cells (*p* < 0.001). These data indicate that ACE2 expression is upregulated in LUAD-derived A549 cells, consistent with prior transcript-level observations in lung cancer tissues. The cytotoxicity of mRBD3dopa-BTZ and free BTZ was assessed in ACE2-high A549 cells and ACE2-low HSAEC cells using the MTT assay at 24 h and 48 h. After incubation for 24 h, free BTZ induced greater cytotoxicity in A549 cells than mRBD3dopa-BTZ. However, after incubation for 48 h, the cytotoxicity of mRBD3dopa-BTZ was significantly higher than that of free BTZ, indicating sustained intracellular delivery and prolonged drug action. These results show that free BTZ rapidly diffuses into cells and acts faster through passive diffusion, while mRBD3dopa-BTZ takes longer to internalize but enhanced effect is attributed to ACE2-mediated endocytosis and pH-triggered BTZ release within endosomes. The IC_50_ values after 48 h were 38.67 ± 1.5 nM for free BTZ and 27.73 ± 1.6 nM for mRBD3dopa-BTZ, demonstrating improved potency of the conjugate over time (Fig. 2*C*).

Since the mRBD3dopa-BTZ conjugate releases active BTZ within the tumor microenvironment, it is expected to act through the same proteasome inhibition mechanism as free BTZ. BTZ, a representative boronic acid proteasome inhibitor, reversibly inhibits the chymotrypsin-like activity of the β5 subunit of the 26S proteasome by interacting with its active-site threonine (39). To directly link the cytotoxic effect to proteasome inhibition, the A549 cells were treated with the different concentrations of free BTZ and mRBD3dopa-BTZ for 48 h, lysed, and proteasome activity was measured using a 20S proteasome activity assay kit. Both treatments significantly reduced proteasome activity in a concentration-dependent manner compared to the untreated control. At 25 nM, mRBD3dopa-BTZ caused significantly greater inhibition than free BTZ (∗∗∗∗*p* < 0.0001), while no significant difference was observed at 35 nM. At 45 nM, proteasome activity decreased to ∼28% of control for mRBD3dopa-BTZ *versus* ∼43% for BTZ (∗∗∗∗*p* < 0.0001). This confirmed that mRBD3dopa-BTZ maintained the inhibitory potency of BTZ (Fig. 2*D*). Live and dead cells were further distinguished using fluorescein diacetate (FDA) and propidium iodide (PI) staining. FDA identifies live cells (green) by measuring cytoplasmic esterase activity, while PI stains dead cells (red) by detecting loss of plasma membrane integrity (Fig. 2*E*).

### Safety and specificity of mRBD3dopa-BTZ conjugate in normal cells

The cytotoxicity of mRBD3dopa-BTZ and free BTZ was evaluated in AC2-low HSAEC cells using the MTT assay. After 48 h, both mRBD3dopa-BTZ and free BTZ maintained >75% viability at all tested concentrations, confirming their high biocompatibility in non-cancerous cells. Notably, mRBD3dopa-BTZ consistently preserved higher cell viability compared to free BTZ across all concentrations (Fig. 3*A*). The non-drug protein components, mRBD3 and mRBD3dopa, exhibited no significant cytotoxicity in A549 cells at concentrations up to 2000 μg/ml over 24 h and 48 h, maintaining cell viability above 85% in all cases (Fig. 3*B*). These findings confirm their safety profile and support their suitability as biocompatible carriers for drug delivery. Furthermore, the mRBD3dopa-BTZ conjugate's safety was verified by assessing its cytocompatibility in human leukemia monocytic cells (THP-1-derived M0 macrophages). At the applied concentration of 35 to 45 nM, neither mRBD3dopa-BTZ nor free BTZ affected the viability of M0 macrophage cells (Fig. 3*C*). This low toxicity aligns with previous reports showing that M0 macrophages remain viable even at free BTZ concentrations as high as 100 μM (40). In contrast, the native SARS-CoV-2 spike protein has been reported to trigger apoptosis in THP-1-like macrophage cells at 100 nM (41). The mRBD3dopa-BTZ conjugate demonstrated no acute toxicity at the lower 35 to 45 nM concentrations, indicating its biocompatibility and supporting its role in enabling targeted and safe drug delivery.

### Cellular uptake and apoptotic mechanism of the ACE2-targeted mRBD3dopa-BTZ in A549 cells

The cellular uptake of fluorescein isothiocyanate (FITC)-labeled mRBD3dopa-BTZ conjugate in A549 cells were examined using fluorescence microscopy. The intense green fluorescence dots dispersed in the cytoplasm indicate that the conjugate's internalization occurs mainly through ACE2-mediated endocytosis (Fig. 3*D*). Next, the link between proteasome inhibition and apoptosis-inducing potential of free BTZ and mRBD3dopa-BTZ in A549 cells is directly correlated with mitochondrial dysfunction. The mitochondrial membrane potential (MMP) assay, an early marker of mitochondrial integrity and apoptosis, was evaluated using two methods: JC-10 and Rho-123 assays. The JC-10 assay measures mitochondrial depolarization by detecting changes in fluorescence ratios, while the Rho-123 assay quantifies it through dye retention. In the JC-10 assay, treatment with mRBD3dopa-BTZ resulted in a concentration-dependent decrease in the JC-10 aggregate/monomer fluorescence ratio, indicating loss of MMP. At 35 nM, the ratio decreased to ∼53% of control for mRBD3dopa-BTZ compared to ∼68% for free BTZ (∗∗∗∗*p* < 0.0001), and at 45 nM it dropped further to ∼34% *versus* ∼48%, respectively (∗∗∗∗*p* < 0.0001), demonstrating greater mitochondrial depolarization by the conjugate. These changes correspond to enhanced green fluorescence, a hallmark of mitochondrial membrane depolarization in apoptotic cells (Fig. 3*E*i). Consistent with these findings, Rho-123 staining showed that both mRBD3dopa-BTZ and free BTZ reduced MMP, as evidenced by decreased red fluorescence relative to the intense red signal in untreated control cells (Fig. 3*E*ii). Together, these results indicate that mRBD3dopa-BTZ induces more pronounced mitochondrial dysfunction than free BTZ at equivalent concentrations, potentially contributing to its enhanced pro-apoptotic activity.

To explore the cellular processes mediating mRBD3dopa-BTZ–induced apoptosis in A549 cells, we examined whether cytochrome c release triggered by the conjugate could activate caspase-9 and caspase-3 in the apoptotic cascade (42). Caspase-9 (initiator caspase) measures the activation of the intrinsic apoptosis pathway, while caspase-3 (executioner caspase) reflects the final execution phase of apoptosis in the apoptotic cascade. As shown in Figure 4*A*, treatment with mRBD3dopa-BTZ at 35 nM and 45 nM produced a significant, concentration-dependent increase in the activity of both caspase-9 and caspase-3 compared to the untreated control (∗∗∗∗*p* < 0.0001, two-way ANOVA with Tukey’s multiple comparisons). Caspase-9 activity rose to ∼140% and ∼160% of control at 35 nM and 45 nM, respectively, while caspase-3 activity increased more markedly, reaching ∼185% and ∼205% of control. These results demonstrate that mRBD3dopa-BTZ exerts its anti-cancer effects *via* a caspase-dependent apoptotic pathway, engaging upstream mitochondrial activation of caspase-9 and downstream execution through caspase-3. To confirm and further characterize this process, annexin V/PI assay was used. This assay measures phosphatidylserine externalization and plasma membrane integrity to distinguish between early and late apoptotic cells in A549 and HSAEC cells. In A549 cells, following 48 h of treatment with mRBD3dopa-BTZ, the population of early apoptotic cells (annexin V+/PI-) significantly increased compared to free BTZ treatment (89.4% *vs.* 37.5%). This increase was then normalized with apoptotic cells in untreated control-viable cells (annexin V−, PI−). On the other hand, the mRBD3dopa-BTZ conjugate demonstrated a lack of ability to induce apoptosis and cell death in HSAEC compared to free BTZ (5.4% *vs.* 36.5%). The observed lack of effect can be attributed to the enhanced efficacy of the conjugate in specifically targeting and killing A549 cells through ACE2-mediated endocytosis and taking advantage of the pH sensitivity of the conjugate, this specificity ensures that normal cells remain unharmed (Fig. 4*B* and Table S7). These results offer insight into the caspase pathway activation, which is involved in both early and late apoptotic processes.

Consistently, apoptotic nuclear morphology was visualized using Hoechst 33342 staining, a DNA-binding dye that detects chromatin condensation and nuclear fragmentation, hallmarks of apoptosis. After 48 h of treatment, the mRBD3dopa-BTZ showed chromatin condensation and nuclear fragmentation comparable to free BTZ, efficiently killing A549 cells (Fig. 4*C*). At this stage, a dose-dependent cascade of events was observed: mRBD3dopa-BTZ inhibits proteasome activity in A549 cells, which was directly correlated with mitochondrial dysfunction, as measured by the loss of mitochondrial membrane potential (ΔΨm) in the JC-10 assay. This mitochondrial dysfunction is a well-established trigger for the permeabilization of the mitochondrial outer membrane and the subsequent release of cytochrome c into the cytosol. The release of cytochrome c promotes the formation of the apoptosome, which drives the activation of the initiator caspase, caspase-9. This, in turn, leads directly to the activation of the executioner caspase-3 and resulting in apoptosis. Together with annexin V/PI staining and nuclear fragmentation assays, these results confirm that mRBD3dopa-BTZ induces apoptosis through a proteasome inhibition-mediated intrinsic caspase-dependent pathway (Fig. S6*E*).

### Comparative evaluation of mRBD3dopa-BTZ efficacy in 3D cancerous and non-cancerous spheroid models

In comparison to 2D models, 3D cultures can provide insights into the impact of tumor microenvironments on the efficacy of anticancer drugs. The two different spheroid models were developed to study the effects of mRBD3dopa-BTZ conjugate on ACE2-high A549 cells and ACE2-low HSAEC cells through receptor-mediated, site-specific delivery. The liquid-overlay method was used to create both the three-dimensional (3D) A549 and HSAEC spheroids (43). After 48 h of treatment, the relative cell viability of 3D A549 and HSAEC spheroids was assessed using the MTT assay. In 3D A549 spheroids, mRBD3dopa-BTZ treatment resulted in significantly lower cell viability compared to free BTZ at all tested concentrations from 50 nM to 200 nM (∗∗∗∗*p* < 0.0001), while no difference was observed at 0 nM. The cytotoxic effect of the mRBD3dopa-BTZ was dose-dependent, reducing viability to ∼25% at 200 nM (Fig. 4*D*i). In contrast, in ACE2-low 3D HSAEC spheroids, the conjugate maintained significantly higher cell viability than free BTZ at all concentrations ≥ 50 nM (∗∗∗∗*p* < 0.0001), indicating reduced toxicity toward healthy cells. Even at the highest concentration (200 nM), viability remained above 70% for mRBD3dopa-BTZ (Fig. 4*D*ii). Quantitatively, the mRBD3dopa-BTZ conjugate significantly reduced the spheroid area in ACE2-high 3D A549 spheroid compared to free BTZ (46% vs. 62%, ∗∗∗∗*p* < 0.0001). This enhanced potency was confirmed by its lower (IC_50_ values of conjugate 125 ± 1 *vs.* free BTZ 181 ± 2 nM). Consistently, spheroid area was preserved at ∼87% relative to the untreated control, demonstrating selective cytotoxicity toward ACE2-high cells while maintaining biocompatibility with normal epithelial cells (Fig. 4*D*iii). The proteasome inhibition was evident at both 125 nM and 150 nM. At 125 nM, both treatments produced comparable inhibition, although mRBD3dopa-BTZ showed a slightly greater reduction (∗∗∗*p* < 0.001). At 150 nM, mRBD3dopa-BTZ reduced proteasome activity to ∼22% of control compared to ∼46% for BTZ (∗∗∗∗*p* < 0.0001). Moreover, the proteasome-inhibiting activity was higher in conjugate-treated A549 spheroid, effectively killing cancer cells with minimal impact on normal cells (Fig. 4*D*iv). Live and dead cells were distinguished by FDA (green) and PI (red) staining (Fig. 4*D*v). An annexin V-FITC/PI assay assessed apoptotic induction in 3D spheroid models. Upon treatment with the mRBD3dopa-BTZ conjugate, the efficacy of BTZ was remarkably enhanced, resulting in an increased percentage of apoptotic cells compared to free BTZ treatment (64.9% *vs.* 39.2%) at 48 h. Conversely, in 3D HSAEC culture, the mRBD3dopa-BTZ conjugate showed a comparatively lower number of apoptotic cells (5.9% *vs.* 22%), a level normalized with the apoptotic cells in the untreated control-viable cells (Fig. 4*E* and Table S8). This suggests that ACE2 receptor-mediated targeting enhances uptake, improves penetration, and increases BTZ accumulation in the tumor site, potentially overcoming BTZ resistance associated with 3D structure and microenvironment of cancer cells.

### *In vivo* antitumor efficacy and safety assessment of mRBD3dopa-BTZ in a syngeneic lung cancer model

The *in vivo* efficacy and safety of BTZ-loaded mRBD3dopa for lung cancer was evaluated using female C57BL/6 mice (aged 6–8 weeks). All procedures were approved by the Institutional Animal Ethical Committee of Central Leather Research Institute, India, and IAEC No.08/2023 (A). A reproducible syngeneic model for lung cancer was the lewis lung carcinoma (LLC) model, in which LLC1 cells derived from C57BL/6 mice are implanted into immunocompetent C57BL/6 hosts. The LLC cell lines are highly tumorigenic and commonly used to model metastasis and evaluate the drug efficacy in *vivo* (44). In this study, a subcutaneous tumor model was established by injecting LLC1 cells (1 x 10^6^ cells/mice) suspended in 0.1 ml of serum-free DMEM into syngeneic C57BL/6 mice (45). When mean tumor volume reached ∼200 mm^3^ on day 14 after inoculation, mice were randomized (n = 8/group) and treated intravenously (i.v.) twice weekly with 1 mg/kg mRBD3dopa, 1 mg/kg free BTZ, or mRBD3dopa-BTZ containing 1 mg/kg BTZ; controls received PBS on the same schedule. The optimal dose and schedule of BTZ was 1.0 mg/kg i.v. given twice weekly, as previously described (46, 47). The LLC1 tumor-bearing lungs in each group were observed and photographed after a 30-days observation period, as shown in (Fig. 5*A*). After treatment begin, mRBD3dopa-BTZ demonstrated greater suppression of tumor growth than both free BTZ and vehicle, with significant differences emerging from ∼day 20 onward (*p* < 0.05, Tukey). The mRBD3dopa carrier alone showed no reduction in tumor progression compared with control, confirming the absence of intrinsic therapeutic action (Fig. 5*B*). In terms of safety metrics, throughout the 30-day period, all treatment groups exhibited steady increases in body weight with no significant intergroup variation, further supporting the good systemic tolerance of the conjugate (Fig. 5*C*). No significant differences were observed in relative liver weight among the groups, indicating the applied doses did not cause evident organ toxicity (Fig. S7*A*). Further, the systemic toxicity was evaluated by measuring serum alkaline phosphatase (ALP) levels. The mRBD3dopa carrier demonstrated intrinsic safety with ALP levels (41.2 ± 3.5 U/L) compared to controls (46.8 ± 3.2 U/L). The free BTZ caused a mild increase in ALP (49.6 ± 3.7 U/L) compared to control (*p* < 0.05 to ∗∗*p* < 0.001), suggesting a moderate hepatobiliary response typically associated with BTZ administration. In contrast, ALP activity in the mRBD3dopa-BTZ group (44.1 ± 3.4 U/L) remained statistically similar to the control and mRBD3dopa groups (*p* > 0.05), confirming that conjugation effectively reduced BTZ-induced hepatic stress (Fig. S7*B*). Overall, these results demonstrate that mRBD3dopa-BTZ administration did not cause appreciable hepatic or systemic toxicity compared with free BTZ.

At the end of the experiment, the tumors and the major organs, such as the lung, liver, kidney and heart, were fixed in paraffin and sectioned into 6-μm-thick pieces for hematoxylin-eosin (H&E) staining. The lung histology of the control (PBS-treated) and mRBD3dopa-treated groups revealed a severe chronic inflammatory state. This morphological change was characterized by epithelial dysplasia in the bronchi, dense peribronchial and interstitial infiltration composed primarily of plasma cells and lymphocytes, and inflammatory cells surrounding the alveoli, while blood vessels remained normal. After treatment with free BTZ and mRBD3dopa-BTZ, the bronchi exhibited normal epithelium, normal blood vessels, and a significant reduction in inflammation to only a mild infiltration composed predominantly of lymphocytes. The mRBD3dopa-BTZ conjugate treatment preserves lung architecture, with minimal residual inflammation and complete resolution of dysplasia, confirming that the conjugate retains the potent anti-inflammatory and anti-dysplastic efficacy of the free drug (Fig. 5*D*).

### Organ-protective efficacy, selectivity, and apoptotic induction of mRBD3dopa-BTZ *in vivo*

The effect of treatments on the three important ACE2-expressing organs, the liver, kidney, and heart, were studied. The liver sections from the control and mRBD3dopa groups displayed normal liver architecture, characterized by well-organized hepatocytes, normal central veins, normal portal triads, and clear sinusoidal spaces. In contrast, free BTZ treatment induced dense periportal inflammation and marked bile duct proliferation within the portal triad, accompanied with necrosis, indicating chemical cholangitis and hepatitis. However, the mRBD3dopa-BTZ conjugate exhibited markedly reduced hepatotoxicity, showing milder periportal inflammation, and bile duct proliferation was still observed in the portal triad. Also, the surrounding hepatic parenchyma generally appeared better preserved. These observations indicate that conjugation substantially mitigates BTZ-induced liver injury. Kidney tissues from the control and the mRBD3dopa groups showed normal glomeruli, healthy renal tubules with intact epithelial lining and clear lumina, and a non-infiltrated interstitium. Free BTZ treatment caused nephrotoxicity, including hypercellular glomeruli with endocapillary proliferation, tubulointerstitial damage, tubular stress, interstitial inflammatory infiltration, and edema. In contrast, the mRBD3dopa-BTZ conjugate maintained a normal glomerular structure with intact tubules and showed a non-inflamed interstitium, and prevented the tubulointerstitial damage observed with free BTZ, confirming protection against nephrotoxicity. Cardiac tissue of all treatment groups demonstrated a normal cardiac morphology, specifically showing normal-appearing cardiomyocytes that were elongated, eosinophilic, and exhibited subtle striations. Similarly, the myonuclei were uniformly intact and normal, presenting as darkly stained, oval, and centrally located within the cells. Importantly, the intercalated disc space was also normal across all samples, appearing as a dense, transverse line between cells, confirming the preservation of cell-to-cell junctions. The mRBD3dopa-BTZ conjugate group showed no obvious difference from the other experimental groups, thus demonstrating a preserved and normal myocardial architecture (Fig. 6*A*). Immunohistochemical analysis was carried out to confirm the apoptosis signaling pathways, focusing on nuclear Factor kappa-light-chain-enhancer of activated B cells (NF-κB) and C/EBP homologous protein also known as DNA damage-inducible transcript 3, DDIT3 (CHOP). In sections of lung tumor tissue, the mRBD3dopa-BTZ conjugate had a stronger impact on causing cell apoptosis, as demonstrated by a much larger CHOP fluorescence area than both free BTZ and the control group. The conjugate was observed to have smaller NF-κB fluorescent regions than control and free BTZ groups with larger NF-κB fluorescent regions (Fig. 6*B*). The elemental boron concentrations in the tissues were evaluated using inductively coupled plasma mass spectrometry (ICP-MS), revealed distinct biodistribution patterns between the free BTZ and mRBD3dopa-BTZ conjugate. In the lung, BTZ accumulation was significantly higher with mRBD3dopa-BTZ compared to free BTZ (36.1 ± 0.63% *vs.* 21.65 ± 0.68%), consistent with its ACE2-targeting design. Conversely, accumulation in other ACE2-expressing organs was reduced in the conjugate group, including the heart (5.68 ± 0.24% *vs.* 9.03 ± 0.53%), kidney (8.07 ± 0.33% *vs.* 13.17 ± 0.61%), and liver (16.12 ± 0.44% *vs.* 27.33 ± 0.76%), indicating enhanced lung targeting while minimizing off-target organ exposure (Fig. 6*C*). BTZ therapeutically inhibits NF-κB activation (48), a pathway often associated with cancer cell survival and resistance to chemotherapy (49). As a result, Western blot quantification demonstrated that both free BTZ and mRBD3dopa-BTZ treatments significantly reduced the expression of the NF-κB compared with the untreated control. The reduction was more pronounced in the mRBD3dopa-BTZ-treated cells, suggesting enhanced intracellular drug retention and potency of the conjugate. As shown in Figure 6*D*, protein bands at 65 kDa were observed, indicating that mRBD3dopa-BTZ targets NF-κB and related cytokine secretion. The densitometry analysis normalized to GAPDH revealed approximately a 2-fold decrease following free BTZ treatment (*p* < 0.01) and a nearly 3-fold decrease with mRBD3dopa-BTZ (*p* < 0.001) compared with the control group. Thus, the mRBD3dopa–BTZ conjugate demonstrated significant improvements in organ-protective efficacy and selective tumor targeting, while also enhancing its anti-tumor potency by promoting apoptosis.

## Discussion

There is a high demand for preparing homogenous protein-drug conjugates for medical applications. However, using protein-based carriers for drug conjugation suffers from several drawbacks, including the potential for structural damage, loss of potency or sufficient selectivity, heterogeneity in the final product, and the inability to achieve specific conjugation sites (50, 51). Current random and non-specific conjugation methods often result in the deactivation of functional proteins, leading to precipitation or unfavorable masking of active sites (52). Protein engineering platforms overcome these limitations by expanding the genetic code to enable precise, site-specific modifications (53). This approach utilizes advances in bio-orthogonal chemistry to produce homogeneous and tunable protein conjugates for diverse applications (54). Notably, the genetic incorporation of unnatural amino acids enables highly precise conjugation, which has been successfully applied in fields such as accelerated wound healing (26).

In this study, we combined the genetic code expansion (GCE) strategy with dynamic covalent boronate chemistry. This enabled us to achieve two main goals: (1) constructing more stable, site-selective, and homogenous conjugates, and (2) functionalizing the protein by incorporating an unnatural amino acid with a unique chemical handle. This chemical handle allows for bio-orthogonal and site-specific drug conjugation, thereby improving drug delivery to lung tumor cells. The versatility of the GCE platform and our site-specific conjugation strategy provided essential tools for targeted delivery. The COVID-19 pandemic highlighted the role of ACE2 in lung physiology and its upregulation in NSCLC (55), which inspired us to exploit ACE2 overexpression as a novel target for receptor-mediated drug delivery in NSCLC cells.

Initially, we rationally designed three distinct RBD variants and introduced a genetic linker DOPA to enhance binding affinity with ACE2. Both RBDs and RBDdopa’s successfully recapitulated the binding and functional activities of the respective wild-type nRBD (Fig. S1). These advances in rational design strategies allow the creation of novel functionalities in proteins by fine-tuning chemical and structural modifications (56, 57, 58).

Although the SARS-CoV-2 RBD expressed in mammalian cells exhibits low expression yield and high cost, the *E. coli* expression system offers easy scalability and reduced low cost (59). The recombinant SARS-CoV-2 RBDs and RBDdopa’s were obtained with high purity and good expression yield using *E.coli* tyrosine auxotroph expression systems (Fig. S2).

The production of recombinant proteins using synthetic biology and recombinant technology is being advanced to: (i) increase expression yield and scalability, (ii) introduce non-canonical amino acids for site-specific bioconjugation, and (iii) improve folding and stability for therapeutic use (60). Currently, the GCE method has been applied in engineering receptor-targeting and multifunctional biomaterials for diagnostics and targeted drug delivery (61, 62, 63).

We successfully incorporated the genetic linker DOPA into RBDs with an incorporation efficiency of more than 90% (Figs. S2 and S3). This site-specific incorporation of the genetic linker DOPA enhanced binding with recombinant ACE2 without altering the secondary structure (Figs. S3–S5). The introduction of catechols through GCE enables their use in bio-sensing, implant coatings, bioconjugation, adhesive materials, biocatalysis, metal chelation, and nano-biotechnology (64).

Recently, DOPA and its analogues were docked into the RBD, showing favorable interaction (65). Various DOPA-based drug carriers have been developed for pH-responsive drug delivery, including DOPA-modified recombinant mussel adhesive protein (34), DOPA-introduced mussel-derived PEP-RGD peptide (66), DOPA conjugated GPLD polypeptides (67), catechol-grafted chitosan-BTZ nanoparticles (68), hyaluronic acid-catechol-BTZ systems (69), and gelatin-DOPA-based nano-gels (70), demonstrating that catechol chemistry can be effectively used for targeted drug delivery.

The key functionality of our final construct, mRBD3dopa, lies in its readily reactive catechol groups with enhanced binding affinity for the ACE2 receptor (Fig. 1*A*). These groups conjugate with the boronic acid-containing BTZ through pH-sensitive dynamic boronate ester chemistry, making them suitable for anticancer-drug delivery (71, 72, 73).

Several BTZ-based drug delivery systems using catechol-boronate ester chemistry, such as BTZ-catechol prodrug micelles (74), boronate-mediated liposomal encapsulation (75), catechol-functional polyelectrolyte complexes (76), BTZ-encapsulated metal-phenolic nanoparticles (77), multifunctional telodendrimer systems (78), and supramolecular nanomedicines (79) have been developed, although many suffer from limitations including lack of conjugation precision, premature drug leakage, poor batch reproducibility, and uncertain pharmacokinetics. We addressed these issues by achieving site-specific incorporation and bio-orthogonal conjugation efficiency above 90%, enabling pH-responsive drug delivery confirmed by UV/Vis spectroscopy, ^11^B NMR, and XPS analysis (Figs. 1, *B* and *F* and 2*A*). The homogeneity of the mRBD3dopa-BTZ conjugate was validated by MALDI-TOF and DLS analysis, confirming residue precision and control over specific BTZ-binding sites (Figs. 1*D* and S6*A*).

Our data showed minimal BTZ release at physiological pH 7.4 (∼7.5% at 48 h) but efficient release under tumor-mimicking acidic conditions (∼70–80% at pH 6.5–5.0) (Fig. 1*E*). RP-HPLC quantification confirmed these values and closely agreed with UV-Vis data (Fig. S6, *B* and *C*). This mechanism enhances drug targeting while minimizing toxic side effects (80, 81).

The conjugate's selectivity was investigated in ACE2-high A549 cells (12, 82) and ACE2-low HSAEC cells (83) using *in vitro* 2D monolayer and 3D spheroid models. The conjugate exhibited significantly higher cytotoxicity than free BTZ in both A549 2D and 3D cultures. These findings can be attributed to the conjugate overcoming the free BTZ’s limitations (low solubility and aqueous instability), thus achieving prolonged cancer cell proliferation inhibition (Figs. 2*C* and 4*D*i). In contrast, mRBD3dopa-BTZ showed higher viability in both HSAEC 2D and 3D cultures compared to free BTZ, indicating minimal off-target cytotoxicity (Figs. 3*A* and 4*D*ii). These findings highlight the conjugate’s selective activity toward ACE2-overexpressing cancer cells while sparing healthy epithelial cells. To quantitatively correlate HPLC-determined BTZ release with pharmacological efficacy, we compared IC_50_ values of the conjugate (27.7 nM in 2D monolayer and 125 nM in 3D spheroid) with free BTZ (38.7 nM and 181 nM, respectively). At physiological pH 7.4, only ∼7.5% release occurred, yielding released concentrations (C_R_) of ∼20.8 nM (2D) and ∼93.8 nM (3D), both below the free BTZ IC_50_, confirming minimal off-target exposure. In contrast, at tumor-acidic pH 6.5, the ∼69.9% release produced C_R_ values of ∼193.8 nM (2D) and ∼873.4 nM (3D), exceeding the free BTZ IC_50_ by ∼5.0-fold and ∼4.8-fold, respectively. These findings confirm that the conjugate confers controlled, pH-responsive release and selectively improves efficacy under tumor-relevant conditions while substantially reducing off-target exposure.

The antitumor effects of proteasome inhibitors primarily involve the blockage of the activation of the NF-κB signaling pathway (the most recognized mechanism of action) (84, 85), and the induction of mitochondrial intrinsic apoptosis through altered mitochondrial membrane permeability leading to apoptotic protein release (86). The mRBD3dopa-BTZ conjugate was expected to act through the same proteasome inhibition mechanism as free BTZ, yet it revealed stronger proteasome inhibition in both 2D and 3D culture (Figs. 2*D* and 4*D*iv).

Using ACE2-high A549 cells, we demonstrated that the conjugate followed the same cascade of events - proteasome inhibition leads to the accumulation of ubiquitinated proteins, endoplasmic reticulum stress, mitochondrial stress, and activation of the intrinsic caspase-dependent apoptotic pathway (Fig. S6*E*).

Our *in vivo* experiments confirmed that the mRBD3dopa-BTZ conjugate showed lower systemic toxicity (as evidenced by ALP levels) and improved selectivity, achieving ∼1.7-fold higher tumor accumulation while reducing distribution to non-target ACE2-expressing organs (liver, kidney, and heart) (Fig. 6, *A* and *C* and Data S7). Furthermore, it produced greater tumor growth inhibition (Fig. 5*B*) and a ∼50% stronger reduction in NF-κB expression (≈3-fold vs. ≈2-fold decrease) by inhibiting the TNFα-induced NF-κB complex. This prevents cancer cells from recovering and repairing damaged intracellular proteins, ultimately leading to apoptosis (Fig. 6, *B* and *D*).

Together, our results confirm that the *in vitro* cell death mechanism correlates with the *in vivo* anticancer effects. Collectively, these findings demonstrate measurable improvements in efficacy, selectivity, and systemic safety, a key goal in the development of targeted conjugates for cancer therapy (87).

In conclusion, this is the first study reporting SARS-CoV-2 RBD-mediated site-specific drug delivery targeting ACE2-overexpressing NSCLC cells. Due to ACE2-mediated endocytosis and pH-triggered drug release, the mRBD3dopa-BTZ conjugate shows great potential as a tumor-targeted proteasome inhibitor for NSCLC treatment. This advancement provides a generalizable, straightforward approach for systematically incorporating amino acids with novel physical, chemical, and biological properties, enabling the creation of similar targeted drug conjugates for metabolic, cardiovascular, and inflammatory diseases. Future work will focus on potential improvements, including increasing drug loading, refining linker chemistry, and enhancing targeting efficiency to further strengthen the translational potential of this system.

## Experimental procedures

### Purification of recombinant RBDs and RBDdopa’s

The recombinantly expressed RBDs, ACE2, and RBDdopa’s cell pellets were lysed using a cell lysis buffer (10 mM Tris-HCl, 1 mM EDTA, 0.1% SDS, and 0.2 mg/ml lysozyme) through sonication. Following the cell lysis, the suspensions were centrifuged at 9000 rcf for 20 min at 4 °C. The protein-soluble fractions were collected and analyzed by 12% SDS-PAGE. Using the AKTA Explorer FPLC system, all the proteins were purified by a Ni-NTA affinity column, which was initially equilibrated with a sodium-phosphate buffer (50 mM sodium dihydrogen phosphate, 20 mM imidazole, 300 mM NaCl, pH 8.0), and elution was achieved through a gradient concentration of imidazole. The purified protein samples with His-tag were desalted using a Sephadex G25 HR column (Cytiva). The protein expression yield was calculated based on the amount of purified protein obtained per liter of *E. coli* culture.

### Circular dichroism (CD) spectroscopy

The secondary structures of proteins (RBDs, RBDdopa’s, and ACE2) with a concentration of 0.3 mg/ml are analyzed using a JASCO J-715 Spectrometer (Jasco UV Co, Ltd) (59, 88). The spectral range extended from 200 to 250 nm. A cuvette with a path length of 0.1 cm was used to record the absorption spectrum, with a scan rate of 50 nm/min at RT. The average spectrum was generated from three individual scans, each with a response time of 1 s, a bandwidth of 1 nm, and a data pitch of 0.1 nm. The raw data were analysed using Jasco software, and the final results were interpreted using Origin software (Origin Lab Corporation).

### Boron nuclear magnetic resonance (B^11^ NMR)

For the Boron NMR, 1 mg/ml of mRBD3dopa-BTZ and free BTZ samples at pH 7.4 and 5.0 were directly examined using B^11^ NMR on a Varian Unity 500 spectrometer, with deuterated D_2_O as the solvent. Boronic acid was used as an external standard for calibration during the analysis.

### X-ray photon spectroscopy (XPS)

The XPS was performed to evaluate the elemental composition of boron present in the mRBD3dopa-BTZ conjugate at both pH 7.4 and 5.0 conditions using a Kratos Axis Ultra DLD spectrometer. Monochromatic Al KR radiation with photon energy (hν) of 1486.58 eV was used, and the high-resolution B 1s element spectra were obtained with a constant analyzer pass energy of 50 eV.

### Proteasome inhibition assay

The chymotrypsin-like proteasome activity in free BTZ and conjugate- treated on both 2D monolayer and 3D multicellular spheroid cell cultures was measured using a 20S proteasome activity assay kit (Sigma Aldrich, cat-APT280). In brief, both spheroids and monolayers were lysed using proteasome activity buffer (composed of 50 mM Hepes at pH 7.5, 5 mM EDTA, 150 mM NaCl, and 1% Triton X-100). Then, the lysates were incubated with the proteasome substrate LLVY peptide bound with fluorophore 7-amino-4-methylcoumarin (LLVY-AMC), which were selectively identified and cleaved by the enzyme 20S proteasome, resulting in the release of the AMC. The emitted free AMC fluorescence was measured using a fluorometer with a 380 nm excitation and a 460 nm emission filter. Control samples consisted of cells only treated with the assay buffer. The proteasome activity was expressed as a percentage relative to the untreated control (0 nM). Experiments were performed using three independent biological replicates, each measured with five technical replicates. Technical replicates were averaged prior to statistical analysis, and the mean value from each biological replicate is displayed as an individual data point in the plot.

### Fluorescence assorted cell sorting

A549 (4 × 10^5^ cells) were seeded into a 6-well plate and allowed to adhere for 24 h. The cells were treated with free BTZ and mRBD3dopa-BTZ conjugate (45 nM) for 48 h, and then washed thrice with 1× binding buffer, followed by the annexin V-FITC apoptosis detection kit guidelines (Sigma Aldrich, cat-APOAF). Eventually, approximately 1 × 10^6^ cells, constituting about 100 μl suspensions, were treated with 5 μl of Annexin V-FITC conjugate and 10 μl of PI and incubated for 15 min in the dark at 37 °C. Then, the volume of the cell suspension was increased to 500 μl by adding binding buffer (1×) and analyzed using a flow cytometer, FACS Aria (BD Biosciences). Gating and analysis were performed using FlowJo software. The experiment was performed in three independent biological replicates, yielding comparable gating profiles across all replicates.

### Cytotoxicity assay for 3D spheroid models

To initiate the treatment, 3-day-old spheroids (both A549 and HSAEC) were moved to a fresh 96-well plate, which had been pre-coated with 1% agarose. Each well received 200 μL of freshly prepared DMEM serum-free medium containing varying concentrations (ranging from 0 to 250 nM) of free BTZ and mRBD3dopa-BTZ. The cultures were then incubated for 48 h. 20 μL of MTT solution was introduced to each well containing A549 and HSAEC cultures and left to incubate for 4 h. Following incubation, the content of each well containing the cultures was transferred to a new flat-bottom 96-well plate, which was then centrifuged at 1000 rcf for 5 min. After centrifugation, 150 μL of media was removed from each well in the culture plates, and 100 μL of DMSO was added. The absorbance was ultimately recorded at 570 nm using an Epoch 2 microplate reader (BioTek Instruments) (89). The concentrations of free BTZ and the mRBD3dopa-BTZ conjugate that led to 50% cell death (IC_50_) in spheroid cultures were determined from the respective dose-response curves. Experiments were performed using three independent biological replicates, each comprising five technical replicates. Technical replicates were averaged prior to statistical analysis, and the resulting biological means are shown as individual data points overlaid on the plot.

### Lewis lung cancer model

Female C57BL/6 mice aged 6 to 8 weeks old and weighing 18 to 22 g were approved by the Institutional Animal Ethical Committee of Central Leather Research Institute, India (IAEC No. 08/2023 (A)). All animals were maintained under sanitary conditions with human care by regular monitoring of body weight, food intake, animal activity, mortality rates, and excretion. Lewis lung carcinoma cells (LLC1) of 1 x 10^6^ cells per mice suspended in 0.1 ml of serum-free DMEM were injected subcutaneously into C57BL/6 mice. The tumor size and body weight were measured using calipers every 2 days. By day 14 post-inoculation, when the average tumor volume reached 200 mm^3^, the mice were divided into four groups (n = 6). Animals in the groups were intravenously (i.v.) administered twice weekly with doses of 1 mg/kg of body weight of mRBD3dopa, mRBD3dopa-BTZ containing 1 mg/kg body weight of BTZ and 1 mg/kg body weight of free BTZ, while the control mice received 1× PBS vehicle on the same schedule. The tumor volume was determined using the formula V = (L × W^2^)/2, where V = volume of the tumor (mm^3^), L = length (mm) and W = width (mm) of the tumor. The mice were sacrificed using CO_2_, the tumor tissues, and the major organs like lungs, heart, liver, and kidney were removed, measured, and analyzed for further studies.

## Data availability

All data are available in the main text or the supplementary materials.

## Supporting information

This article contains supporting information. Experimental procedures, methods and all other information are all provided in the supporting text, Figs. S1–S7 and Tables S1–S8. The authors have cited additional references within the supporting information (90, 91, 92, 93, 94, 95, 96, 97, 98, 99, 100, 101, 102, 103, 104, 105, 106, 107, 108).

## Conflict of interest

The authors declare that they have no conflicts of interest with the contents of this article.
