# Supplementary material for: Catechol-linker and receptor-mediated site-specific delivery of bortezomib against non-small cell lung cancer
Source: J Biol Chem. 2025 Dec 22;302(2):111095. doi: 10.1016/j.jbc.2025.111095 (PMC12828769; doi:10.1016/j.jbc.2025.111095)
Supplement: Supporting information [file mmc1.docx]

**Supporting Information**

**Catechol-Linker and Receptor mediated Site-specific Delivery of Bortezomib against Non-Small Cell Lung Cancer**

*Mohan Indhu,^[a],[b]^ Valappil Sisila,^[a],[b],[d]^  Devandran Jayasurya,^[c]^ Niraikulam Ayyadurai*^[a],[b]^*

^[a]^ Department of Biochemistry and Biotechnology, Council of Scientific and Industrial Research (CSIR) - Central Leather Research Institute, Chennai, Tamil Nadu – 600020, India

^[b]^ Academy of Scientific and Innovative Research (AcSIR), Ghaziabad-201002, India

^[c]^ Department of pharmacology, The Tamil Nadu Dr. M.G.R. Medical University, Chennai, Tamil Nadu – 600032, India

^[d]^ Present address: Department of Chemistry, Syracuse University, Syracuse, New York 13244, United States

***Correspondence to: Dr N Ayyadurai**
E-mail: [ayyadurai@clri.res.in](mailto:ayyadurai@clri.res.in) & [ayyadurai@gmail.com](mailto:ayyadurai@gmail.com)

**Materials.**

Promega in Madison, WI, USA, provided the PCR reagents, T4 DNA ligase, and restriction endonucleases. Isopropyl-β-D-thiogalactopyranoside (IPTG), lysozyme, L-3,4-dihydroxyphenylalanine (DOPA), Nitroblue tetrazolium (NBT), Bortezomib (BTZ) and all the necessary chemicals for the experiments were purchased from Sigma in St. Louis, USA. Preparation of plasmid DNA using the host bacterium Escherichia coli (*E. coli*) strain XL1-blue from Strata Gene, USA. Nickel nitrilotriacetic acid (Ni-NTA) affinity column was obtained from Clontech, Takara Bio, USA. The plasmid pQE-80L was acquired from Qiagen in Valencia, USA, and the E. coli strain JW2581 tyrosine auxotroph was procured from the E. coli Genetic Stock Centre at Yale University, USA. Capillaries for microscale thermophoresis (MST) were purchased from NanoTemper Technologies, Germany. Human lung carcinoma epithelial cells (A549, CRM-CCL-185), Human leukemia monocytic cell line (THP-1, TIB-202), Primary Small Airway Epithelial Cells; Normal, Human (HSAEC, PCS-301-010), and Lewis lung carcinoma cell line from the lung of a C57BL/6 mouse (LLC1, CRL-1642) cells were procured from American Type culture collection (ATCC), Washington DC, USA. Dulbecco's modified eagle medium (DMEM) high glucose, Roswell park memorial institute (RPMI) 1640 medium with L-glutamine, 10% Fetal Bovine Serum (FBS), 1X antibiotic/antimycotic solution, Trypsin- Ethylenediamine tetraacetic acid (EDTA), MTT (3-[4,5-dimethylthiazol-2-yl]-2,5 diphenyl tetrazolium bromide), WST-1 (2-(4-Iodophenyl)-3-(4-nitrophenyl)-5-(2,4-disulfophenyl)-2H-tetrazolium), Total RNA Isolation Reagent (TRIzol), Fluorescein diacetate (FDA), Propidium iodide (PI), DAPI (4',6-diamidino-2-phenylindole), Hoechst-33342, Hoechst 33258, Mitochondrial Membrane Potential (MMP) detection kit, rhodamine-123 (Rho-123), Annexin V-FITC Apoptosis Detection Kit, caspase-3 and caspase-9 assay kit colorimetric, FITC (Fluorescein isothiocyanate), Deuterated Dimethyl sulfoxide-D6 (DMSO-d6), phorbol-12-myristate13-acetate (PMA), Bovine serum albumin (BSA), Protease and Phosphatase inhibitors, RIPA lysis buffer were procured from Sigma, St. Louis, USA. Anti-GAPDH antibody [6C5] (Abcam; Cat-ab8245), GAPDH Rabbit polyclonal antibody (Abcam; Cat-ab22555), Primary Antibodies NF-kB p65 Mouse Monoclonal Antibody-1:200 in PBST (Affinity Biosciences; Cat-BF8005), CHOP Mouse Monoclonal Antibody-1:100 in PBST (abbkine; Cat-ABM40322), ACE2 Rabbit polyclonal antibody (Abcam; Cat-ab15348), Secondary Antibody-Goat Anti-Mouse IgG (H+L) Fluor647-conjugated (Affinity Biosciences; Cat-S0014)-1:200 in PBST, Horseradish peroxidase (HRP)-conjugated goat anti-rabbit IgG (H+L) secondary antibody (Jackson ImmunoResearch, USA; Cat-AB2307391) were used. Additionally, 10% bovine serum albumin (BSA), Tris-buffered saline with 0.1% Tween-20 (TBST), Triton X 100 – 500 µL + 1X PBS – 5 mL (PBST), 100% Ice cold Methanol, 1X phosphate buffered saline (1X PBS), bicinchoninic acid (BCA) assay kit (Thermo Fisher Scientific, USA), polyvinylidene difluoride (PVDF) membranes (Millipore, USA), Blocking agent (10% C57BL/6 mice serum in 1X PBST), RNase A (100µg/ mL), Glycerol, and Agarose were employed in the experiments.

**Experimental procedures.**

**Rational designing of SARS COV-2 RBD.**

The amino acid sequences of nRBD, mRBD1, mRBD2 and mRBD3 are assembled in the following way to get the corresponding sequences:

- nRBD - Native SARS-CoV-2 RBD (Arg319-Phe541) without any mutation

RVQPTESIVRFPNITNLCPFGEVFNATRFASVYAWNRKRISNCVADYSVLYNSASFSTFKCYGVSPTKLNDLCFTNVYADSFVIRGDEVRQIAPGQTGKIADYNYKLPDDFTGCVIAWNSNNLDSKVGGNYNYLYRLFRKSNLKPFERDISTEIYQAGSTPCNGVEGFNCYFPLQSYGFQPTNGVGYQPYRVVVLSFELLHAPATVCGPKKSTNLVKNKCVNF

- mRBD1 - the four serine residues are mutated rationally to threonine

RVQPTE**T**IVRFPNITNLCPFGEVFNATRFASVYAWNRKRISNCVADYSVLYNSA**T**FSTFKCYGVSPTKLNDLCFTNVYADSFVIRGDEVRQIAPGQTGKIADYNYKLPDDFTGCVIAWNSNNLDSKVGGNYNYLYRLFRK**T**NLKPFERDISTEIYQAG**T**TPCNGVEGFNCYFPLQSYGFQPTNGVGYQPYRVVVLSFELLHAPATVCGPKKSTNLVKNKCVNF

- mRBD2 - In addition to the four serine mutations, 2 glycoacylation sites of ASN 331,342 mutated to serine and threonine

RVQPTE**T**IVRFP**S**ITNLCPFGEVF**T**ATRFASVYAWNRKRISNCVADYSVLYNSA**T**FSTFKCYGVSPTKLNDLCFTNVYADSFVIRGDEVRQIAPGQTGKIADYNYKLPDDFTGCVIAWNSNNLDSKVGGNYNYLYRLFRK**T**NLKPFERDISTEIYQAG**T**TPCNGVEGFNCYFPLQSYGFQPTNGVGYQPYRVVVLSFELLHAPATVCGPKKSTNLVKNKCVNF

- mRBD3 - In addition to the four serine mutations and two asparagine mutation, RBD domain has more number of tyrosine so the structurally unaffected tyrosine residues were mutated with structurally similar amino acids

RVQPTE**T**IVRFP**S**ITNLCPFGEVF**T**ATRFASVYAWNRKRISNCVAD**W**SVLYNSA**T**FSTFKCYGVSPTKLNDLCFTNVYADSFVIRGDEVRQIAPGQTGKIAD**F**NYKLPDDFTGCVIAWNSNNLDSKVGGNYNYLYRLFRK**T**NLKPFERDISTEI**F**QAG**T**TPCNGVEGFNC**H**FPLQSYGFQPTNGVG**W**QPYRVVVLSFELLHAPATVCGPKKSTNLVKNKCVNF

- Human Angiotensin-converting enzyme 2 (ACE2)

QSTIEEQAKTFLDKFNHEAEDLFYQSSLASWNYNTNITEENVQNMNNAGDKWSAFLKEQSTLAQMYPLQEIQNLTVKLQLQALQQNGSSVLSEDKSKRLNTILNTMSTIYSTGKVCNPDNPQECLLLEPGLNEIMANSLDYNERLWAWESWRSEVGKQLRPLYEEYVVLKNEMARANHYEDYGDYWRGDYEVNGVDGYDYSRGQLIEDVEHTFEEIKPLYEHLHAYVRAKLMNAYPSYISPIGCLPAHLLGDMWGRFWTNLYSLTVPFGQKPNIDVTDAMVDQAWDAQRIFKEAEKFFVSVGLPNMTQGFWENSMLTDPGNVQKAVCHPTAWDLGKGDFRILMCTKVTMDDFLTAHHEMGHIQYDMAYAAQPFLLRNGANEGFHEAVGEIMSLSAATPKHLKSIGLLSPDFQEDNETEINFLLKQALTIVGTLPFTYMLEKWRWMVFKGEIPKDQWMKKWWEMKREIVGVVEPVPHDETYCDPASLFHVSNDYSFIRYYTRTLYQFQFQEALCQAAKHEGPLHKCDISNSTEAGQKLFNMLRLGKSEPWTLALENVVGAKNMNVRPLLNYFEPLFTWLKDQNKNSFVGWSTDWSPYADQSIKV

**Bioinformatics.**

To understand the binding property of RBDs (both the RBDs without modification and genetically modified RBDs) with ACE2, the structures are predicted for a rationally designed sequence of RBDs using alphafold, followed by visualizing the RBDs, highlighting the tyrosine residues and thereby DOPA incorporation by replacing tyrosine using PyMOL. The pLDDT score for all the predicted structures is greater than 65, indicating high reliability of the proteins. The symmetry-related dimer of the models was developed using two methodologies: one employing a protein-protein protocol with the ClusPro server and the other using the Rosetta ROSIE server. The model was chosen based on the lowest symmetry score.

**Cloning and recombinant expression of RBDs and ACE2.**

The constructs pQE80L-nRBD, pQE80L-mRBD1, pQE80L-mRBD2 and pQE80L-mRBD3 (RBDs-pQE80L) were transformed into *E. coli* auxotrophic strains-Tyr auxotroph (JW2581). Then, the transformed *E. coli* tyrosine auxotroph strain JW2581 was cultured in 10 mL of initial luria-bertani (LB) medium supplemented with ampicillin (100 µg/ mL) at 37 ºC and 180 rpm. From this, 1% of primary cells were inoculated into 1 L of LB broth containing ampicillin (100 µg/ mL) and cultivated until the early mid-log phase (OD_600_, 0.5-0.6) at 37 ºC. The recombinant RBDs and ACE2 production were optimized using 1 mM isopropyl β-D-thiogalactopyranoside (IPTG) at various induction conditions, production temperature (18 ºC, 25 ºC, 30 ºC, and 37 ºC), production time (2, 4, 6, and 18 h), and IPTG induction concentration (0.2, 0.4, 0.6, 0.8, 1, and 2 mM). The culture was then centrifuged at 10,000 rpm for 10 min to separate the cell pellet from the supernatant. The samples were run through 12% sodium dodecyl sulphate polyacrylamide gel electrophoresis (SDS-PAGE).

**Residue-specific incorporation of genetic linker DOPA.**

The introduction of DOPA residues at specific sites in *E. coli* JW2581 strains containing pQE80L plasmids encoding sequences for nRBD, mRBD1, mRBD2, and mRBD3 was followed using the outlined protocol(26). RBDs-pQE80L in *E. coli* tyrosine auxotroph for targeted DOPA incorporation(90, 91) were cultured in LB broth containing ampicillin and incubated at 37 °C for 12 h. After harvesting, the cultures were transferred to minimal media (MM) containing 20% glucose, 20 amino acids, 1 mM CaCl_2_, 1 mM MgSO_4_, thiamine HCl (1mg/ mL), and ampicillin (100 µg/ mL) and incubated at 37°C for 6 to 12 h. Followed by a 15 min centrifugation at 5000 rpm and thorough washing in 1X PBS, the cells were re-inoculated into 20% glucose minimal media (MM) containing 20% glucose, 19 amino acids, supplemented with a minimal amount of 0.03 mM tyrosine (Tyr) and ampicillin (100µg/ mL). The cultures were grown until reached the mid-log phase (OD_600_ 0.6-0.8) at 180 rpm, 37 °C. At this phase, 1 mM DOPA was introduced to the culture flask before induction with IPTG. To achieve the optimal expression of DOPA-incorporated RBDs, including native RBD (nRBDdopa) and mutant RBD variants (mRBD1dopa, mRBD2dopa, and mRBD3dopa) using residue-specific incorporation techniques, a 1 mM IPTG induction was used and incubated for 6 h at 37 °C. After which, the cells were harvested, and the purification process was carried out.

**RBD-ACE2 receptor binding studies by microscale thermophoresis (MST).**

The changes in binding kinetics of RBDs and RBDdopa’s with ACE2 receptor were studied using MST. ACE2 receptors were labelled with NT-647 dye, forming crosslinks with lysine residues in proteins via N-hydroxysuccinimide ester chemistry. Following the labeling reaction, samples were passed through a Sephadex G-25 desalting column to remove unreacted dye molecules and recover the labeled protein. The ligand-receptor (RBD-ACE2) interactions were probed using the Monolith NT.115 equipment from Nano Temper Technologies, Germany. The experiments were carried out in triplicate, employing standard glass capillaries provided by Nano Temper Technologies. ACE2 receptors undergo serial dilution across 16 tubes using 10 mM phosphate buffer at pH 7.4. For every series of reactions 10 µL of labeled RBDs and RBDdopa’s were added. The reaction mixtures (1:1) were incubated at room temperature (RT) for 15 min before being transferred to MST capillaries. All these studies on protein-receptor binding were conducted at 40-60% MST power(92). The dissociation constant (k_D_) was determined from MO binding affinity Nano Temper Analysis 1.2.231 software. The k_D_ binding model equation based on the Langmuir binding isotherm(93, 94). The equation is as follows:

$$f\left( c \right)= Unbound+\left( Bound-Unbound \right) x c+c \left( \mathrm{receptor} \right)+k_{D}-\frac{\sqrt{c + c (receptor) + kd)^2 ) - 4c x c (receptor)}}{2c \left( \mathrm{receptor} \right)}$$

Where; f(c) = fraction bound at a known ligand concentration c, k_D_ = dissociation constant, and c (receptor) = final receptor concentration in the assay. The unbound and bound are the normalized fluorescence signals of receptor alone and protein‐receptor complex, respectively.

**Matrix-Assisted Laser Desorption Ionization-Time of Flight (MALDI-TOF) analysis.**

Initially, a C18 zip-tip (Millipore, USA) was used for desalting the purified protein samples to eliminate contaminants. Further, after desalting, RBDs, RBDdopa’s and ACE2 were subjected to MALDI-TOF analysis using a sinapinic acid matrix on a Microflex LT instrument (Bruker Daltonik, Germany). This instrument was operated in a linear mode, selected for optimal resolution with a 25 kV extraction voltage and a 13 kV reflectron potential.

**Nitro blue Tetrazolium (NBT) staining.**

The incorporation of DOPA into RBDs and the dopaquinone formation were confirmed by a redox staining assay using nitro blue tetrazolium (NBT) solution. The purified proteins were confirmed using SDS-PAGE, and then were transferred to a polyvinylidene difluoride membrane (PVDF) that had been activated with methanol. The protein bands on the membrane were immersed in a NBT staining solution (2 M sodium glycinate, 0.24 mM NBT, pH 10) in the dark for 3 h. The membrane was then rinsed in a sodium borate solution overnight to improve the visibility of protein bands.

**Preparation of protein-drug conjugates.**

The boronic acid-containing anticancer drug bortezomib (BTZ) was bio-orthogonally conjugated to the proteins mRBD3 (without the genetic linker DOPA) and mRBD3dopa (containing 10 DOPA residues). Each protein was prepared at a concentration of 1 mg/ mL in 1X PBS (pH 7.4), and separately mixed with BTZ at a molar ratio of BTZ/protein of 10:1, ensuring bio-orthogonal conjugation of BTZ was dependent on the available DOPA residues in the protein. The reactions were stirred at RT for 12 h, and then the free BTZ was separated from mRBD3-BTZ and mRBD3dopa-BTZ conjugates against 1X PBS at pH 7.4 using an Amicon ultrafilter unit with a 10 kDa MWCO (Sigma Aldrich) with centrifugation at 4000 rcf for 10 min, repeated three times. The resulting mRBD3-BTZ and mRBD3dopa-BTZ conjugates were again purified using FPLC, using a HiPrep desalting column packed with Sephadex G-25 resin, and then used for further studies.

**Characterization of protein-drug conjugates.**

**UV-Vis Spectroscopy.**

UV-Vis spectroscopy (SCINCO MEGA 800, South Korea) was used to confirm the lambda shift in the protein samples before and after BTZ conjugation(95, 96). The spectral analysis covered a wavelength range of 200 to 800 nm, and the protein concentration for each sample was maintained at 1 mg/ mL.

**CD, MALDI-TOF and MST.**

Protein samples (mRBD3, mRBD3dopa and mRBD3dopa-BTZ) before and after drug conjugation was characterized by CD, MALDI-TOF and MST analysis following the previously outlined procedure. The spectra were plotted using Origin Software (Origin Lab Corporation, USA).

**Dynamic Light Scattering (DLS).**

The evaluation of protein aggregation and conjugate homogeneity was performed in 1X PBS at pH of 7.4, with a constant temperature of 25°C, using a Zetasizer Nano ZS90 instrument (Malvern Instruments in Germany)(97). A 1 mL disposable sizing cuvette (Sarstedt, Germany) was used for the measurements, and the data were analysed using Zetasizer software version 7.12 and plotted using Origin Software (OriginLab Corporation, USA).

**pH-dependant *in vitro* drug release study.**

To study the pH-dependent drug release by an equilibrium dialysis method, the purified BTZ-loaded mRBD3dopa complex (1 mg/ mL, the molar ratio of BTZ/mRBD3dopa is 10:1) was transferred into a dialysis bag (10 kDa), sealed, and then placed in a larger vessel containing approximately 30 mL of release media (1X PBS buffer solutions at each pH value of 7.4, 6.5, and 5.0) at 37 °C with continuous stirring at 300 rpm. The BTZ was released from the mRBD3dopa diffused through the dialysis membrane into the outer compartment. At different time intervals, 500 µL buffer solution was collected from the outer phase and replaced with 500 µL of fresh buffer solution. The collected samples were then analyzed using UV-visible spectroscopy at 267 nm for BTZ to calculate the drug release levels. The cumulative release of the drug was calculated and plotted against release time.

Initially, the burst release kinetics was examined with frequent sampling in the initial hours (every 15 or 30 min). Once the initial burst declines, the sampling intervals are extended to every 1-2 h. The sampling duration is then extended to study the complete drug release profile. Due to the stability of the mRBD3dopa-BTZ conjugate for 3 days at 37 ºC, this sampling duration of 10 h is further extended to examine the sustained drug release systems.

**Quantification of BTZ release by RP-HPLC.**

The BTZ content was quantitated by RP-HPLC (C18, 250 × 4.6 mm, 5 μm; shimadzu), methanol:water 50:50 v/v, 1.0 mL·min⁻¹, as described previously(98). To evaluate acid-triggered hydrolysis of BTZ, 1 mg mRBD3dopa-BTZ was dissolved in 1 mL release media (methanol/PBS, v/v = 60/40, pH 7.4, 6.5 and 5.0) and then incubated at 37 °C with gentle shaking in a shaker. At required time points of 0-60 h, aliquots (n = 3), the released BTZ were separated from mRBD3dopa-BTZ by immediately transferring into a molecular weight cutoff filter of 10 kDa using ultracentrifugation at 4000 rcf for 10 min. The higher molecular weight mRBD3dopa stayed in the supernatant, and the concentration of released BTZ settled down at the bottom was measured by HPLC with the following release profile conditions (pH value of 7.4, 6.5, and 5.0). The sample was eluted at a flow rate of 1.0 mL/ min at RT and detected at 270 nm. The standard curve for BTZ was: y_(BTZ)_ = 114691x – 151363, conducted at a BTZ concentration range of 0-400 µM and yielded linear fits (R² = 0.9976). The cumulative release of the drug was calculated and plotted against release time.

**Validation of cell death mechanism.**

**Cell culture.**

All the cell lines, such as human lung carcinoma epithelial cells (A549, CRM-CCL-185), normal human primary small airway epithelial cells (HSAEC, PCS-301-010), human leukemia monocytic cell line (THP-1, TIB-202), and lewis lung carcinoma cell line from the lung of a C57BL/6 mouse (LLC1, CRL-1642), were procured from the American Type Culture Collection (ATCC), Washington DC, USA. A549 and LLC1 cell lines were cultivated in Dulbecco’s modified eagle medium (DMEM) high glucose supplemented with 10% fetal bovine serum (FBS) and 1X antibiotic/antimycotic solution. HSAEC were maintained in airway epithelial cell basal medium (PCS-300-030, ATCC) supplemented with epithelial small airway cell growth kit components (PCS-301-040, ATCC). THP-1 were cultivated in Roswell Park Memorial Institute (RPMI) 1640 medium with L-glutamine, 10% FBS, and 1X antibiotic/antimycotic solution. All these cell lines were maintained in humidified air at 37 ºC with 5% CO_2_. The culture medium was changed every two to three days, and the cells were sub-cultured regularly using a 0.05% trypsin and 0.02% EDTA solution.

**Analysis of ACE2 gene expression profile with cancer genomics database**.

ACE2 Gene expression was analysed in Lung Adenocarcinoma (LUAD) and Lung Squamous Cell Carcinoma (LUSC) subtypes of NSCLC compared with normal lung tissue using publicly available cancer genomics databases, such as the Cancer Genome Atlas (TCGA) or the genotype-tissue expression (GTEx) project, accessed through an online GEPIA2 platform. The gene expression values were quantified as transcripts per million (TPM), and the expression data were then log_2_ ​transformed after adding a pseudocount of one, as indicated by the y-axis label: Expression=log_2_​(TPM+1). Box plots were generated to visualize the distribution of the log_2_​(TPM+1) expression levels. The analysis compared gene expression in tumor (T) samples versus normal (N) samples within both the LUAD and LUSC cohorts.

**Cytotoxicity analysis of free BTZ and the conjugate in A549 cells.**

A549 cells were detached using a trypsin-EDTA solution for 2 min, following a previously outlined protocol as described(99). The cells were seeded into 96 well plates at a density of 2x10^3^ cells per well and allow to attach for 24 h in complete medium (DMEM high glucose medium with 10% FBS and 1% antibiotic-antimycotic solution). Then, the cells were treated with free BTZ and mRBD3dopa-BTZ conjugate at various concentrations ranging from 0 to 100 nM. The treatments were diluted in serum-free medium (DMEM high glucose with 1% antibiotic-antimycotic solution) and incubated with the cells for 24 h and 48 h time period. The treatment was given once at the start of the experiment, and the treatment medium was not changed during the 24 h and 48 h incubation periods to simulate continuous exposure to a single dose of drug and conjugate without re-dosing. The untreated cells were kept as a control (0 nM concentration). At the end of each treatment period (24 h and 48 h), the treatment medium was removed and replaced with serum-free medium containing [3-(4,5-dimethylthiazol-2-yl)-2,5-diphenyltetrazolium bromide](https://www.google.com/search?sca_esv=ad4fc6a40c0579bc&sxsrf=AE3TifMWB2VzvrKcOHDMcRbefGcolvifmQ%3A1765199023656&q=3-%284%2C5-dimethylthiazol-2-yl%29-2%2C5-diphenyltetrazolium+bromide&sa=X&sqi=2&ved=2ahUKEwj6_ozmhq6RAxUz2DgGHfCDLZAQxccNegQIZRAC&mstk=AUtExfCIBeRrEhuU5mcJzIQJ01SJjF5tCa3VA1_hC34wu5EJxqtJi4oNUbajCuu5NU0LdN8hGQP6q7W3n3jSMPlBY2vlAWlrX1UjOiGeWJnJkz0INTH9SOZpGSJ1lNNRmpUjHfGSlqnm_3SyEHobA8PhKNkhHfTmJ4GvOtFQsGSgjH7ShxTnaiseBsua4HPiYWqKdLk4xdpGdBwR_y1lRyv4PCwe7TaiO6iwHcWhv1jFlROMAkLPpmHFmKEIzGbl1xssa7t4bvS_cDUh1zCWVctCPfjE&csui=3) (MTT, 1 mg / mL) and was added to each well. Then, the cells were incubated for 4 h at 37 °C to allow for the formation of purple formazan crystals. After dissolving the crystals in 100 μL of dimethyl sulphoxide (DMSO), the absorbance was measured at 570 nm using an Epoch 2 microplate reader (BioTek Instruments, USA). The percentage of cell viability was calculated from the recorded absorbance values, which were normalized to the untreated control group. Experiments were conducted in three independent biological replicates, each with five technical replicates. Technical replicate values were averaged prior to statistical analysis, and the mean value of each biological replicates is shown as an individual data point overlaid on the plot.

**Cell viability assay in HSAEC cells.**

HSAEC were trypsinized for 2 min using trypsin-EDTA and seeded into 96-well plates at 2x10^3^ cells per well. After 24 h attachment in complete medium, the cells were treated with free BTZ and mRBD3dopa-BTZ at concentrations of 0 to 100 nM prepared in serum-free medium and incubated for 24 h and 48 h. The treatment was given once at the start of the experiment, and the treatment medium was not changed during the 24 h and 48 h incubation periods to simulate continuous exposure to a single dose of drug and conjugate without re-dosing. The untreated cells were kept as a control (0 nM concentration). At the end of each treatment period (24 h and 48 h), the treatment medium was removed and replaced with serum-free medium containing MTT (1 mg / mL) and was added to each well. Then, the cells were incubated for 4 h at 37 °C to allow for the formation of purple formazan crystals. After dissolving the crystals in 100 μL of DMSO, the absorbance was measured at 570 nm using an Epoch 2 microplate reader (BioTek Instruments, USA). The percentage of cell viability was calculated from the recorded absorbance values, which were normalized to the untreated control group. Experiments were performed using three independent biological replicates, each comprising four technical measurements. Technical replicates were averaged before statistical analysis, and the resulting biological means are displayed as individual data points on the plot.

**Cytotoxicity analysis of carrier proteins in A549 cells.**

To assess cytotoxicity of the carrier proteins, A549 cells were seeded in 96-well plates at 2x10^3^ cells per well following trypsinization. The cells were grown in complete medium for 24 h and treated with proteins (mRBD3 and mRBD3dopa) at concentrations ranging from 0 to 2000 μg/ mL. Protein solutions were prepared in serum-free medium and the treatment was given once at the start of the experiment. The treatment medium was not changed during the 24 h and 48 h incubation periods to simulate continuous exposure to a single dose of proteins without re-dosing. The untreated cells were kept as a control (0 nM concentration). At the end of each treatment period (24 h and 48 h), the treatment medium was removed and replaced with serum-free medium containing MTT (1 mg / mL) and was added to each well. Then, the cells were incubated for 4 h at 37 °C to allow for the formation of purple formazan crystals. After dissolving the crystals in 100 μL of DMSO, the absorbance was measured at 570 nm using an Epoch 2 microplate reader (BioTek Instruments, USA). The percentage of cell viability was calculated from the recorded absorbance values, which were normalized to the untreated control group. Experiments were carried out using three independent biological replicates, each measured in four technical replicates. Technical measurements were averaged before statistical analysis, and the mean value from each biological replicate is displayed as an individual data point on the plot.

**Live/dead staining for 2D A549 cells.**

A549 cells were seeded at a density of 4x10^3^ cells per well and incubated for 24 h before treatment. The anti-proliferative effects of free BTZ and mRBD3dopa-BTZ conjugate were validated through fluorescence microscopic analysis after 48 h of treatment. Then, the old media was removed from the plates, and cells were incubated with the FDA/PI solution for 5 min. The cells were washed with 1X PBS and visualized using a fluorescence microscope (Leica DMI8, Germany) with FITC (green colour) and Rhodamine (red colour) filters. The untreated cells as controls, prior to staining, FDA and PI solutions were prepared at concentrations of 10 µg/ mL and 1 µg/ mL, respectively.

**Evaluation of cytotoxicity on THP-1 derived macrophages.**

THP-1 cells were seeded in 96-well plates at a density of 8000 cells per well, and PMA at a concentration of 100 ng/ mL was added to RPMI serum-free medium to induce cell attachment. Upon PMA treatment, THP-1 cells undergo differentiation into macrophages, as previously described(40). During the differentiation phase, the cells adhered to the flask surface, displaying a change in morphology from spherical to nearly spindle-shaped, along with the development of pseudopods in some cases. Following a 24 h differentiation period into macrophages, after three washes, macrophages were incubated with standard medium containing FBS for 48 h. After resting, both free BTZ and mRBD3dopa-BTZ conjugate concentrations of 35 nM and 45 nM were treated for 48 h to evaluate their effects on cell viability. 10 𝜇L of WST-1 (2-(4-iodophenyl)-3-(4-nitrophenyl)-5-(2,4 disulfophenyl)-2H-tetrazolium, monosodium salt) were added to 100 𝜇L of culturing medium per wells and were incubated for 3 h at 37°C in 5% CO_2_ to allow color conversion of the tetrazolium salt due to mitochondrial activity. Cell viability was determined by measuring the absorbance at 450 nm using Epoch2 microplate reader (BioTek, USA). Experiments were performed using three independent biological replicates, each consisting of six technical replicates. Technical replicate values were averaged prior to statistical analysis, and the mean value of each biological replicate is shown as an individual data point overlaid on the plot.

**Nuclear condensation study.**

The apoptotic induction in A549 cells was examined using Hoechst-33342 stain(100). Cells were seeded at a density of 4x10^3^ cells per well and incubated for 24 h. Then, cells were exposed to various concentrations (0, 35 nM, and 45 nM) of free BTZ and conjugate and, cultured for 48 h under standard conditions. Untreated cells kept as controls. After removing the culture media, the cells were washed with 1X PBS and then fixed in 4% paraformaldehyde for 10 to 15 min, and re-washed twice with 1X PBS. Then the cells were stained with Hoechst-33342 (10 µg/ mL) and incubated for 10 min at 37ºC in the dark. The excess stains were removed by washing with 1X PBS and observed in an inverted fluorescence microscope (Leica DMi8, Germany) with a DAPI (blue colour) filter. Image analysis was performed using Leica Application Suite 4.7 (Leica, Munich, Germany).

**Mitochondrial Membrane Potential (MMP):**

The MMP assay was used to evaluate the early stages of apoptosis in A549 cells by monitoring the initial changes in mitochondrial membrane redox potential. Cells ranging from 2x10^4^ to 8x10^4^ per well in a 96-well plate and were treated with varying concentrations (0, 35 nM, and 45 nM) of both free BTZ and the mRBD3dopa-BTZ conjugate for a duration of 48 h. The JC-10 dye loading solution was prepared according to the Mitochondria Membrane Potential Kit's protocol (Sigma Aldrich, cat. MAK159). Using a spectrophotometer (Thermo Scientific, Varioskan Flash), the samples were measured at 540 and 590 nm for green fluorescence and at 490 and 525 nm for red fluorescence. The ratios of green to red fluorescence were then employed to analyze the rate of apoptosis. Experiments were performed in three independent biological replicates, each containing five technical replicates. Technical replicate values were averaged prior to statistical analysis, and the mean value from each biological replicate is shown as an individual data point overlaid on the plot.

**Rhodamine 123 staining.**

Changes in mitochondrial membrane stability after treatment with free BTZ and conjugate were evaluated through Rhodamine-123 staining (Rh-123)(101, 102). A549 cells were seeded in a 6-well plate at a density of 1x10^5^ cells per well and incubated for 24 h before being subjected to the respective treatments. After 48 h of treatment, the cells were washed with 1X PBS and fixed with 4% paraformaldehyde for 10 min. After fixation, the cells were washed twice with 1X PBS, and then stained with Rh-123 (10µg/ mL) and 4′, 6-diamidino-2-phenylindole (DAPI) (1 µg/ mL) for 30 min at 37 ºC. Then, the excess stain was removed by washing with 1X PBS, and changes in ∆Ψ_m_ were observed using a fluorescence microscope with DAPI (blue) and Rhodamine (red) filters.

**Caspase activity assay.**

The caspase activities in A549 cells were carried out using caspase-9 (APT173) and caspase-3 (CASP3C-1KT) colorimetric assay kits from Sigma Aldrich (St. Louis, MO, USA). After 48 h of treatment with 35 and 45 nM mRBD3dopa-BTZ conjugate, 3×10^6^ cells were lysed using 50 µL of cold lysis buffer and incubated on ice for 10 min. The cell lysate was centrifuged at 10,000 rcf at 4 ℃ for 1 min, and the supernatant was transferred to each well of a 96-well plate with equal volumes of reaction mixture containing 10 mM dithiothreitol (DTT). The DEVD-pNA substrate (4 mM) was added to each well and incubated for 10 min. The absorbance was measured at 405 nm, and the results were represented as the percentage of increase in caspase activity compared to the untreated control (0 nM). Experiments were conducted in three independent biological replicates, each with five technical replicates. Technical replicate values were averaged prior to statistical analysis, and the mean value of each biological replicate is shown as an individual data point overlaid on the plot.

**Preparation of protein-FITC conjugates for cellular uptake study.**

The mRBD3dopa-BTZ was labelled by incubating with amine-reactive FITC at 37 ºC in the dark for 1 h, following a previously outlined procedure(103). Initially, 100 µg of FITC was dissolved in 0.1% DMSO and sequentially added to 1 mg of proteins in Tris buffer (pH 7.4). The FITC-labelled mRBD3dopa-BTZ was separated from free FITC using a 10 kDa cut-off filter (Millipore, USA). Additionally, unbound FITC was also removed by FPLC using a Sephadex desalting column. The mRBD3dopa-BTZ-FITC was excited using a green-colour filter during fluorescence spectrometric analysis. The FITC conjugation and purification procedure was reproduced in three independent protein batches, and similar elution profiles and fluorescence labeling efficiencies were observed across preparations.

**Cellular uptake determined by fluorescence microscopy.**

A549 cells were seeded in 12-well plates at a density of 2×10^5^ cells/ well, 24 h prior to the experiments. The cells were treated with different concentrations of FITC-labelled mRBD3dopa-BTZ conjugate (0, 35, 45, 55 nM) in serum-free medium for a duration of 6 h. The images were captured using an inverted fluorescence microscope (Leica DMi8, Germany) with bright-field and FITC (green) filters. The acquired images were captured using Leica Application Suite 4.7 (Leica, Munich, Germany). Three independent biological replicates were analyzed, and five randomly selected non-overlapping fields were imaged for each replicate.

**Establishment of three-dimensional (3D) spheroid models.**

The three-dimensional (3D) spheroids were generated using the liquid overlay technique in a flat-bottom 96-well plate. Initially, the plate was coated with a 1% (w/v) agarose to create a non-adherent platform for spheroid culture. An ACE2 highly expressing A549 and an ACE2 non-overexpressing HSAEC spheroid culture were established by seeding 1x10^4^ cells in 96-well plates with an agarose bottom. Spheroids were maintained in complete medium and were then incubated at 37 °C and 5% CO_2_ for 3 days without disturbance to facilitate spheroid formation. The images of the spheroids were obtained by the inverted fluorescence microscope (Leica DMi8, Germany). The measurement of spheroid area was calculated using ImageJ software, and the results were compared with the area of spheroids in their respective control groups without treatments. Experiments were conducted in three independent biological replicates, each with six technical replicates. Technical replicate values were averaged prior to statistical analysis, and the mean value of each biological replicate is shown as an individual data point overlaid on the plot.

**Live/dead staining for 3D spheroid models**

The live and dead staining of 3D spheroid models was performed using A549 and HSAEC spheroids. Three-day-old spheroids were transferred into a fresh 96-well plate pre-coated with 1% agarose. Each well received 200 μL of freshly prepared DMEM serum-free medium containing varying concentrations (ranging from 0 to 250 nM) of free BTZ and mRBD3dopa-BTZ and then incubated for 48 h. After treatment, the anti-proliferative effects were assessed using FDA and PI staining. The untreated spheroids are kept as controls. The old media was removed, and spheroids were incubated with FDA and PI staining solution for 5 min at RT. The spheroids were washed with 1X PBS and visualized using a fluorescence microscope (Leica DMI8, Germany) with bright field, FITC (green colour) and Rhodamine (red colour) filters.

**Flow cytometric analysis for 3D spheroid models.**

After both free BTZ and conjugate treatment (125 nM), the spheroid cells were resuspended using trypsin-EDTA solutions and incubated at 37ºC for 2 min to ensure proper cell dissociation. Then, 2 mL of DMEM was added to the mixture and centrifuged at 1000 rpm for 5 min. The cells were then washed with 1X PBS and also re-suspended again in 1X PBS to prevent re-aggregation. Annexin V-FITC/PI staining was carried out using the manufacturer's protocol, and the analyzed cells were examined using Flow cytometer software (BD-FACS). The experiment was performed in three independent biological replicates, yielding comparable gating profiles across replicates.

**Histopathology.**

After the treatment, the main organs that expressed ACE2, such as liver, kidney, lung, and heart, were removed and preserved in a 10% neutral buffered formalin solution. Then, the tumours and organs were embedded in paraffin and sliced into sections with a thickness of 6 µm for hematoxylin and eosin (H&E) staining. The resulting slides were examined using an optical microscope (TS100, Nikon). The staining procedure was performed on tissue samples obtained from three independent biological replicates, yielding consistent tissue morphology and staining intensity across samples.

***In vivo* bio-distribution study*.***

To assess the bio-distribution of BTZ, mice bearing tumours were randomly divided into two groups, each consisting of six mice. A single injection of free BTZ and mRBD3dopa-BTZ conjugate, both administered at a dosage of 1 mg/ kg of body weight, was administered into the right flank of tumour-bearing mice, with 0.9% saline serving as the control. After 12 h, the mice were euthanized, and major tissues such as the heart, kidney, liver, and lung were collected, washed with 0.9% saline, and weighed. Subsequently, all tissues were dissolved in concentrated nitric acid (65% v/v) through heating. The boron content in the resulting solutions was determined using inductively coupled plasma mass spectrometry (ICP-MS), and the BTZ contents in each tissue were then calculated. The results were represented as the percentage of boron concentration in tissues relative to the total injected boron(37). For each treatment group, tissue samples from six independent biological samples were processed and digested separately. Each digest was analyzed in technical triplicate, and technical readings were averaged to obtain a single value per biological replicate. Statistical analyses were performed on the resulting biological replicate means (n = 6).

**Immuno-histochemical analyses.**

At the end of treatments, lung tumor tissues were collected and sliced into 5-μm sections, evaluated by immune-histochemical staining for CHOP, also known as DNA damage-inducible transcript 3 proteins (DDIT3) and nuclear factor kappa-light-chain-enhancer of activated B cells (NF-κB). The 5 μm tissue sections were deparaffinized and rehydrated using three changes of xylene followed by three changes of a graded series of ethyl alcohol. Heat-induced antigen retrieval was performed with 0.1 M citrate buffer, pH 6.0, for 20 min at 100 °C to unmask the antigen epitopes. Antigen-retrieved lung sections from C57BL/6 mice were fixed with 100% ice-cold methanol for 20 min and then incubated with blocking agent (10% mice serum in 1X PBST) for 30 min, followed by twice PBS washes for 5 min. Then the primary antibodies (NF-κB p65 Mouse Monoclonal Antibody-1:200 in PBST and CHOP Mouse Monoclonal Antibody-1:100 in PBST) were added and incubated overnight at 4 °C. Again, the sections were rinsed with PBS twice for 5 min and incubated with secondary antibody (Antibody-Goat Anti-Mouse IgG (H+L) Fluor647-conjugated-1:200 in PBST) for 1 h in the dark. The sections were then incubated with RNase for 30 min at 37 °C, followed by two PBS washes, each for 5 min. Furthermore, it was incubated with DAPI (1:1000 in RNase solution) for 15 min and then mounted using Glycerol. Image acquisition was performed using a LEICA Confocal Microscope (LEICA Microsystems, Heidelberg, Germany). The emission for Fluor647 ranged from 650 to 670 nm and from 350 to 450 nm for DAPI. The NF-κB and CHOP fluorescence intensity was then quantified by normalization to DAPI fluorescence intensity. Representative images were taken from at least three independent biological samples per group.

**Western blotting.**

**Detection of ACE2 expression:** ACE2 expressing cell samples (A549 and HSAEC) were homogenized in RIPA cell lysis buffer (SKU: AR0105-100; Boster Bio, CA, USA) supplemented with protease and phosphatase inhibitors and maintained on ice for 30 min. The lysates were centrifuged at 12,000 × g for 10 min at 4 °C, and the supernatant was collected. Protein concentrations were determined using the bicinchoninic acid (BCA) assay kit (Thermo Fisher Scientific, USA). Equal amounts of protein (30–50 µg) were separated by SDS-PAGE and transferred onto polyvinylidene difluoride (PVDF) membranes (Millipore, USA). Membranes were blocked with 10% bovine serum albumin (BSA) prepared in Tris-buffered saline with 0.1% Tween-20 (TBST) for 1 hour at RT. The membranes were then incubated overnight at 4 °C with primary antibodies against ACE2 (Rabbit polyclonal antibody) and GAPDH (Rabbit polyclonal antibody, used as a loading control). After three washes with TBST, membranes were incubated with appropriate horseradish peroxidase (HRP)-conjugated secondary antibodies (Jackson ImmunoResearch, USA) for 1 hour at RT. Protein bands were detected using enhanced chemiluminescence (ECL) substrate and visualized on X-ray film (Kodak, USA) using manual film development techniques. Band intensities were quantified using ImageJ software (NIH, USA). Quantified band intensities represent mean ± SD from three independent biological replicates, each with three technical replicates. Technical replicate values were averaged prior to statistical analysis, and the mean value of each biological replicate is shown as an individual data point overlaid on the plot. Signal intensities were normalized to GAPDH.

**Detection of NF-ĸB inhibition:** Frozen lung tissues weighing between 0.2 to 0.25 g were homogenized using a pestle homogenizer. 1 mL of lysis buffer, composed of 0.5% NP-40 and 0.5% sodium deoxycholate in a 10 mM Tris buffer at pH 7.5, was introduced. The homogenate was left on ice to swell for 10 min. After centrifugation at 1000 ×g for 8 min, the supernatant was collected and treated with lysis buffer (10 mM Tris–HCl at pH 7.5) containing 50 mM NaCl, 50 mM NaF, 10 mM EDTA, 1 mM DTT, 1% Triton X-100, 0.1% SDS, 1% sodium deoxycholate, 1 mM phenylmethylsulfonyl fluoride, 5 mM leupeptin, and 10 mg/ mL aprotinin for 30 min on ice. The lysates were then centrifuged to eliminate insoluble components and normalized based on their protein content. Debris and nuclei-cleared lysates were resolved on 10% gels. Equal quantities of proteins (30 μg) underwent separation via SDS-PAGE and were transferred onto a PVDF membrane (Millipore, USA). Following a 1 h blocking period at RT using PBST buffer containing 3% (m/v) BSA, the membrane was incubated with anti-NF-κB p65 (diluted 1:1500) and the secondary antibody (diluted 1:2000). The film was developed, and bands corresponding to NF-κB activation were observed. Band intensities were quantified using ImageJ software (NIH, USA). Quantified band intensities represent mean ± SD from three independent biological samples (n = 3), normalized to GAPDH. Representative western blot images are shown.

**Statistical analysis**:

The statistical tests performed, number of replicates, and error measures for each experiment are indicated in the figure legends. All *in vitro* assays were repeated three times. Student’s *t*-test (2-tailed) was used to compare differences between the two groups; One-way ANOVA with Tukey’s post hoc test was used for comparisons among multiple groups, and two-way ANOVA was used for evaluating the effects of two independent variables. All data are expressed as mean ± standard deviation (SD) from at least three independent biological replicates, each done in technical replicates. The data points representing the mean of each independent biological replicate are overlaid. Statistical significance was assessed using the *p*-value (**p* < 0.05; **p < 0.01; ****p* < 0.001; ****p < 0.0001). All data were analyzed using GraphPad Prism 10.1.0 (316) (GraphPad Software Inc., USA).

**RESULTS**

**Figures**


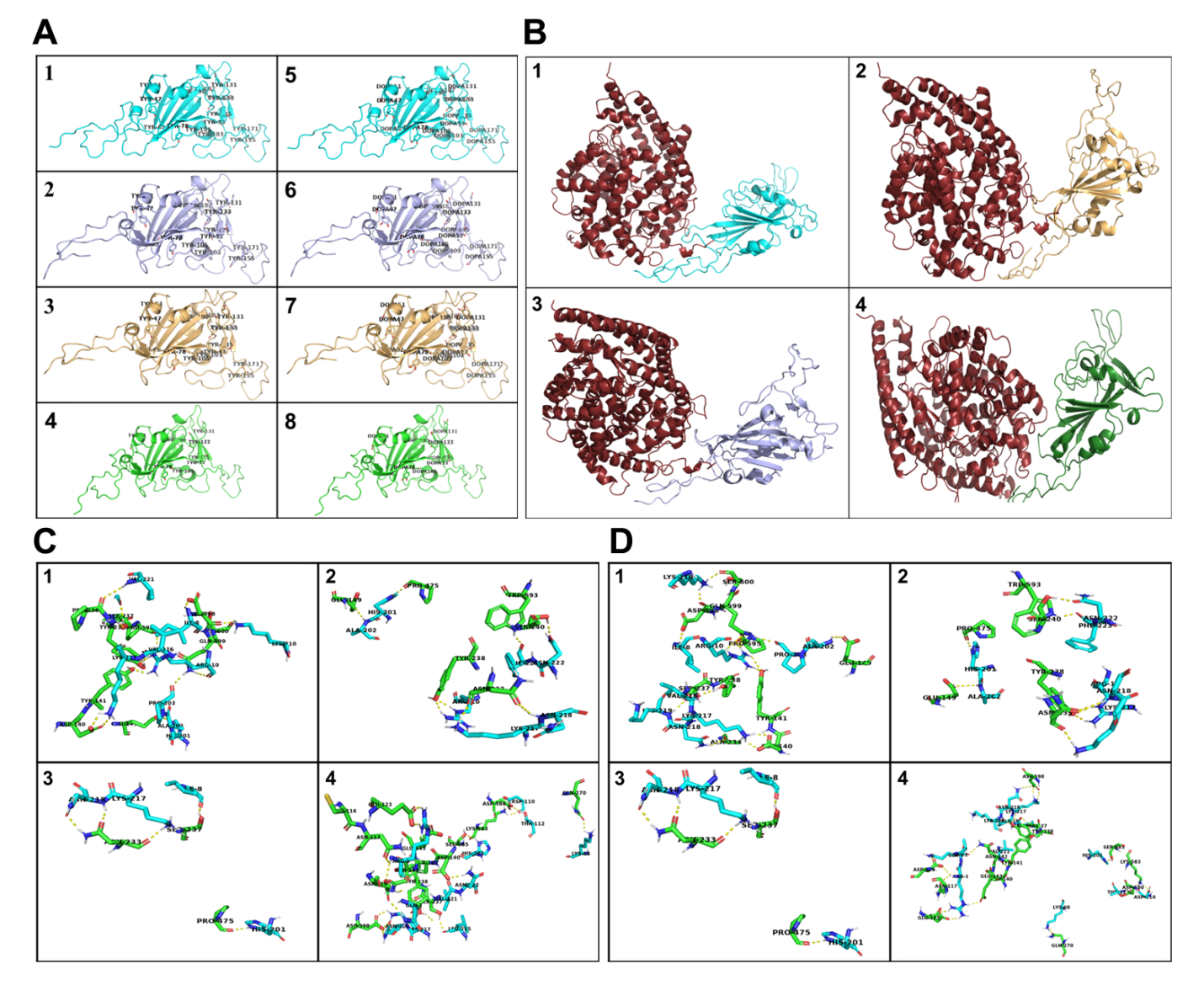


**Figure S1. Structural stability and molecular docking studies.** A, Structure of RBDs and RBDdopa’s using AlphaFold, (1) nRBD, (2) mRBD1, (3) mRBD2, (4) mRBD3, (5) nRBDdopa, (5) mRBD1dopa, (6) mRBD2dopa, and (7) mRBD3dopa. The pLDDT score for all the predicted structures is greater than > 65, indicating that the reliability of proteins is high. B, Docking of RBDs and RBDdopa’s with ACE2, (1) nRBD and nRBDdopa (cyan), (2) mRBD1 and mRBD1dopa (light blue), (3) mRBD2 and mRBD2dopa (light orange) and (4) mRBD3 and mRBD3dopa (green) with ACE2 (red). (C) Interacting AA residues present in (1) nRBD, (2) mRBD1, (3) mRBD2, and (4) mRBD3 are shown in blue colour, and interactions with ACE2 are shown in green. D, Interacting amino acids residues present in (1) nRBDdopa, (2) mRBD1dopa, (3) mRBD2dopa and (4) mRBD3dopa are shown in blue colour interacting with ACE2, shown in green colour.

**
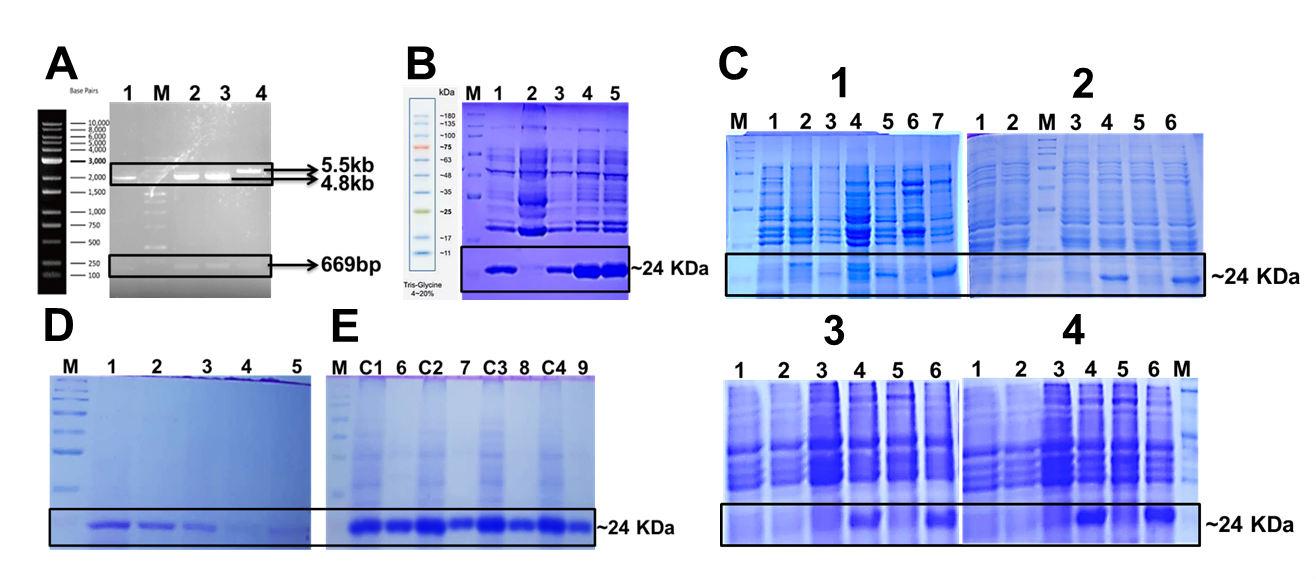
**

**Figure S2. Ribosomal synthesis of RBDs and RBDdopa’s by expanding the genetic code.** A, Restriction digestion analysis of the extracted plasmids, Lane M: 10 kb Marker, Double-digested band of Lane 1: mRBD1dopa in pQE80L, Lane 2: mRBD2dopa in pQE80L, Lane 3: mRBD3dopa in pQE80L, Lane 4: nRBDdopa in pET102. B, Recombinant expression profile of RBDs-pQE80L in *E. coli* tyrosine auxotroph, Lane M: Marker, Lane 1: nRBD, Lane 2: uninduced (without IPTG), Lane 3: mRBD1, Lane 4: mRBD2, Lane 5: mRBD3. C, Evaluating the expression profile for genetic incorporation of DOPA in RBDs (1) nRBDdopa, (2) mRBD1dopa, (3) mRBD2dopa and (4) mRBD3dopa in *E.coli* tyrosine auxotroph, Lane M: marker, Lane h: *E.coli* Tyrosine auxotroph, Lane 1: 19 amino acids without IPTG, Lane 2: 19 amino acids with IPTG, Lane 3: 20 amino acids without IPTG, Lane 4: 20 amino acids with IPTG, Lane 5: DOPA incorporated RBDs without IPTG, Lane 6: DOPA incorporated RBDs with IPTG. D, Purification of RBDs, Lane 1: nRBD, Lane 2: mRBD1, Lane 3: mRBD2, Lane 4, 5: mRBD3. E, Purification of DOPA incorporated RBDs; Lane C1: crude nRBDdopa, Lane 6: purified nRBDdopa, Lane C2: crude mRBD1dopa, Lane 7: purified mRBD1dopa, Lane C3: crude mRBD2dopa, Lane 8: purified mRBD2dopa, Lane C4: crude mRBD3dopa. Lane 9: purified mRBD3dopa.


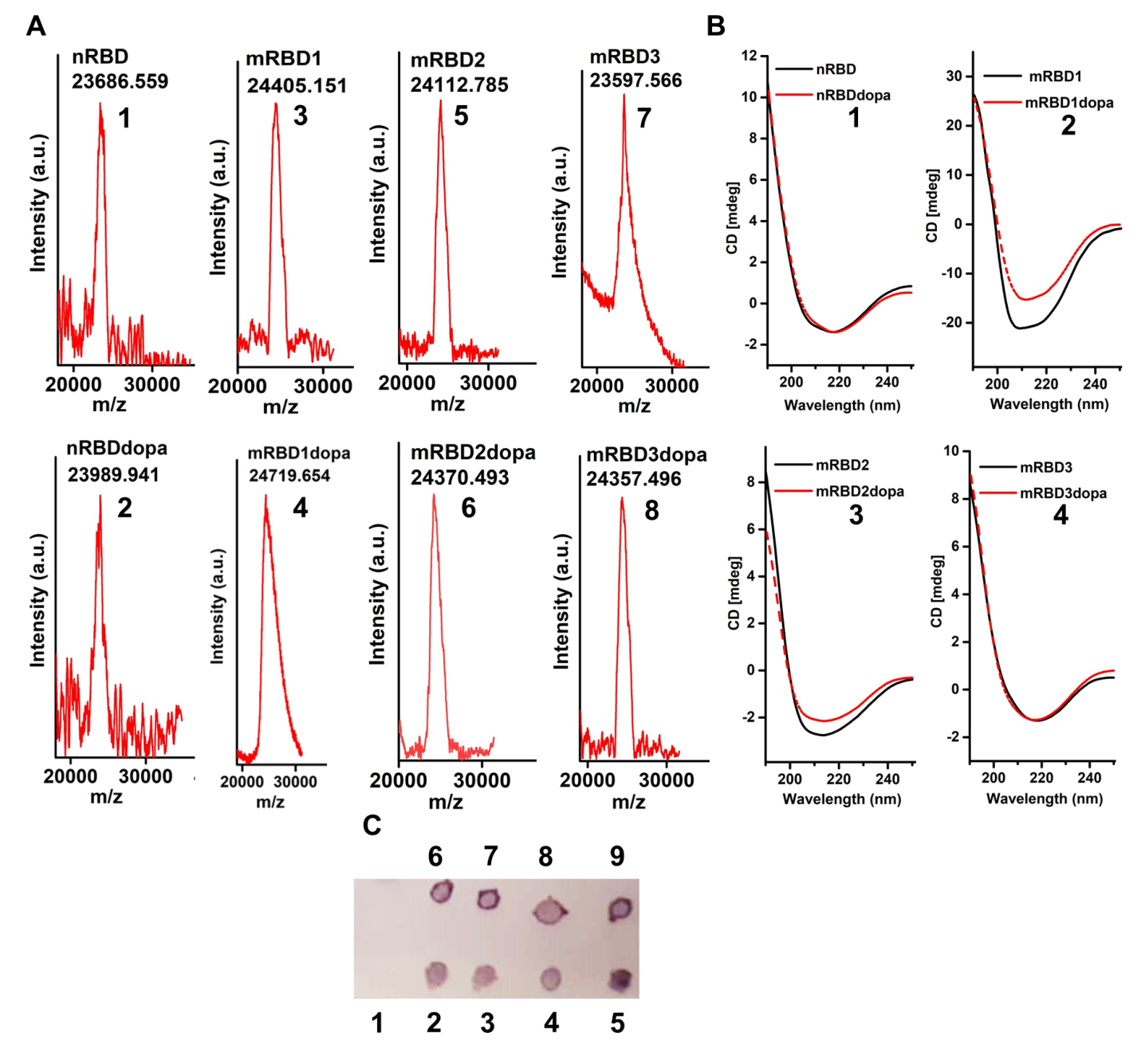


**Figure S3. Biophysical characterization of RBDs and RBDdopa’s.** A, MALDI-TOF analysis and the representative spectra acquired using a sinapinic acid matrix in linear mode, (1, 2) nRBD and nRBDdopa, (3, 4) mRBD1 and mRBD1dopa, (5, 6) mRBD2 and mRBD2dopa, and (7, 8) mRBD3 and mRBD3dopa. B, Representative CD spectrum of (1) nRBD and nRBDdopa, (2) mRBD1 and mRBD1dopa, (3) mRBD2 and mRBD2dopa, and (4) mRBD3 and mRBD3dopa. C, Quinone protein detection assay, the representative image are shown, (1) Tyrosine, (2-5) DOPA, (6) nRBDdopa, (7) mRBD1dopa, (8) mRBD2dopa, and (9) mRBD3dopa.


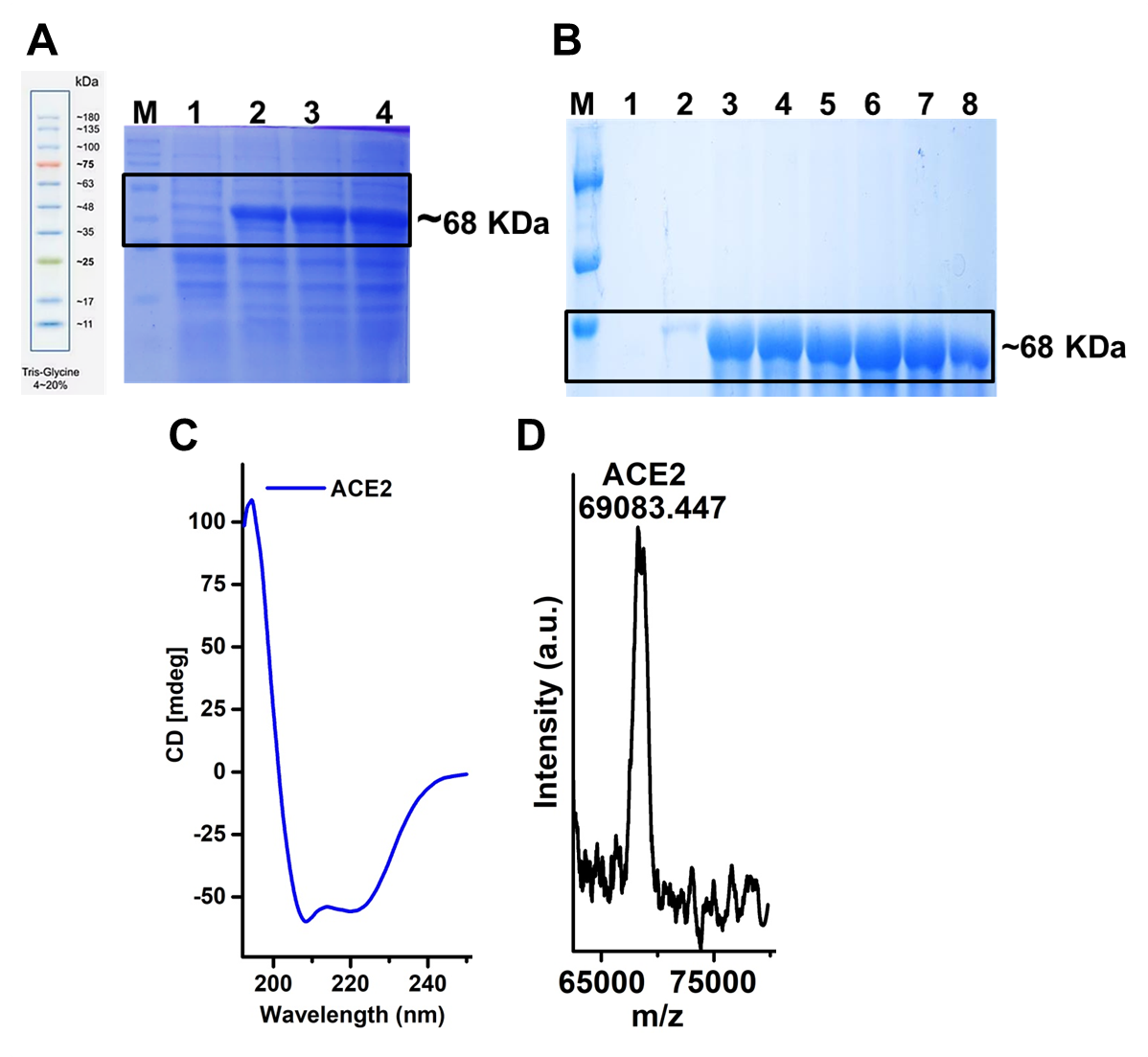


**Figure S4. Production of recombinant ACE2 for binding kinetics analysis.** A, Recombinant expression profile of ACE2-pET102 in *E. coli* BL21(DE3). B, SDS-PAGE analysis of purified recombinant ACE2, Lane M: Marker, Lane 1-8: eluted fractions of purified ACE2. C, Representative CD spectrum of ACE2. D, MALDI-TOF analysis of ACE2 and the representative spectra acquired using a sinapinic acid matrix in linear mode.

**
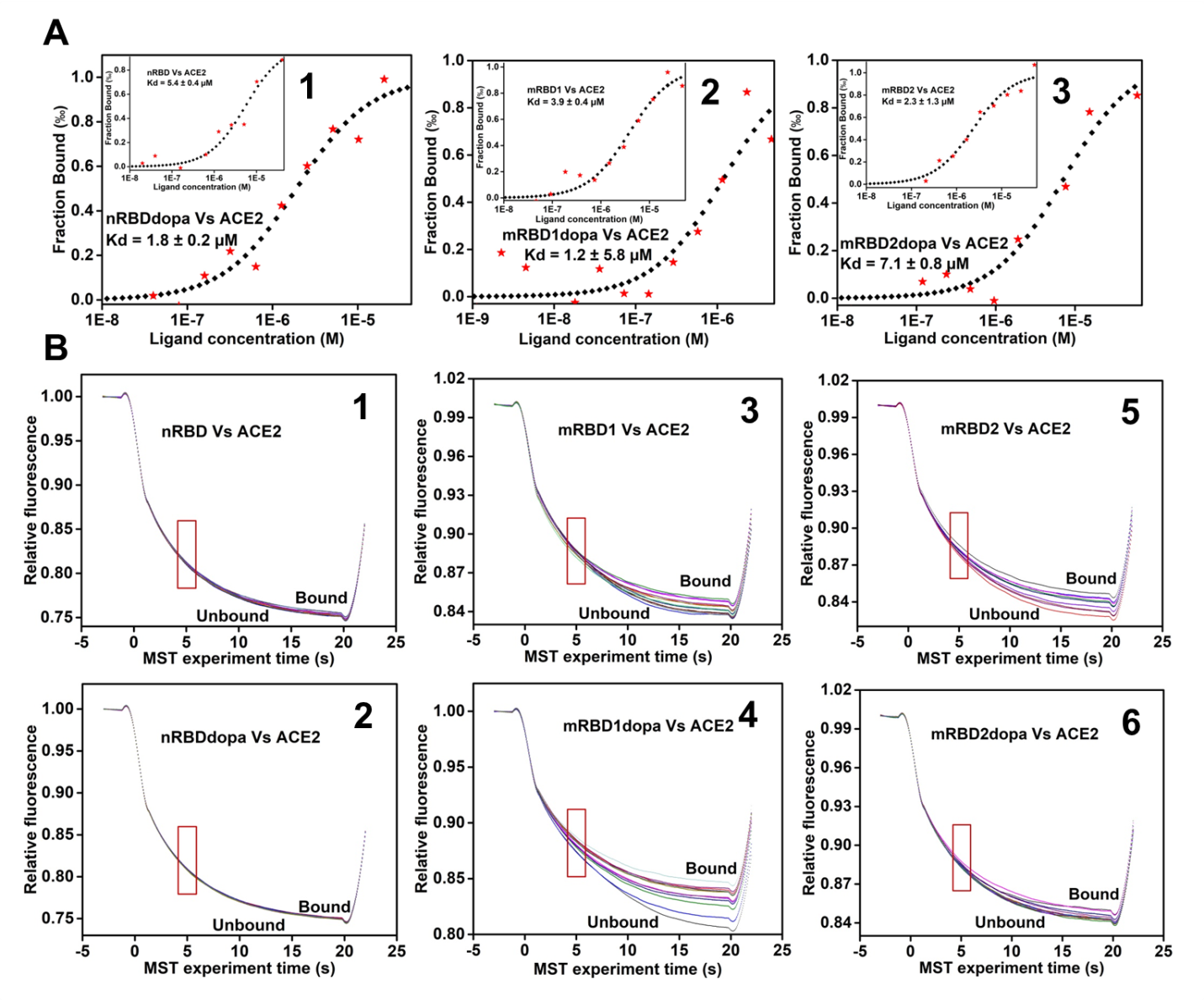
**

**Figure S5. Binding kinetics of RBDs and RBDdopa’s with ACE2.** A, Interaction of recombinant RBDs and RBDdopa’s with ACE2 receptors analysed in 10 mM phosphate buffer using MST, (1) nRBD and nRBDdopa, (2) mRBD1 and mRBD1dopa and (3) mRBD2 and mRBD2dopa with ACE2. The black square indicates the curve generated by Monolith binding affinity Nano Temper Analysis 1.2.231 software, and the red dot indicates the fluorescent intensity (fraction bound) on protein‐receptor interaction. K_D_ values are reported as mean ± SD using three independent protein preparations, each measured in technical triplicate. The binding curves shown are representative of three independent ligand preparations and labeling reactions. B, Thermal activation by the IR laser, the changes in the fluorescence signal occurred in the MST capillaries and ended within a few seconds are denoted by temperature jumps (T jumps) plot of RBDs and RBDdopa’s vs. ACE2 receptor, (1) nRBD, (2) nRBDdopa, (3) mRBD1, (4) mRBD1dopa, (5) mRBD2 and, (6) mRBD2dopa. Initially, constant fluorescence was observed, indicating homogeneous distributions followed by fluorescence changes upon thermal activation, which were recorded for 30 sec. Consequently deactivation of the IR laser led to inverse T-jumps.

**
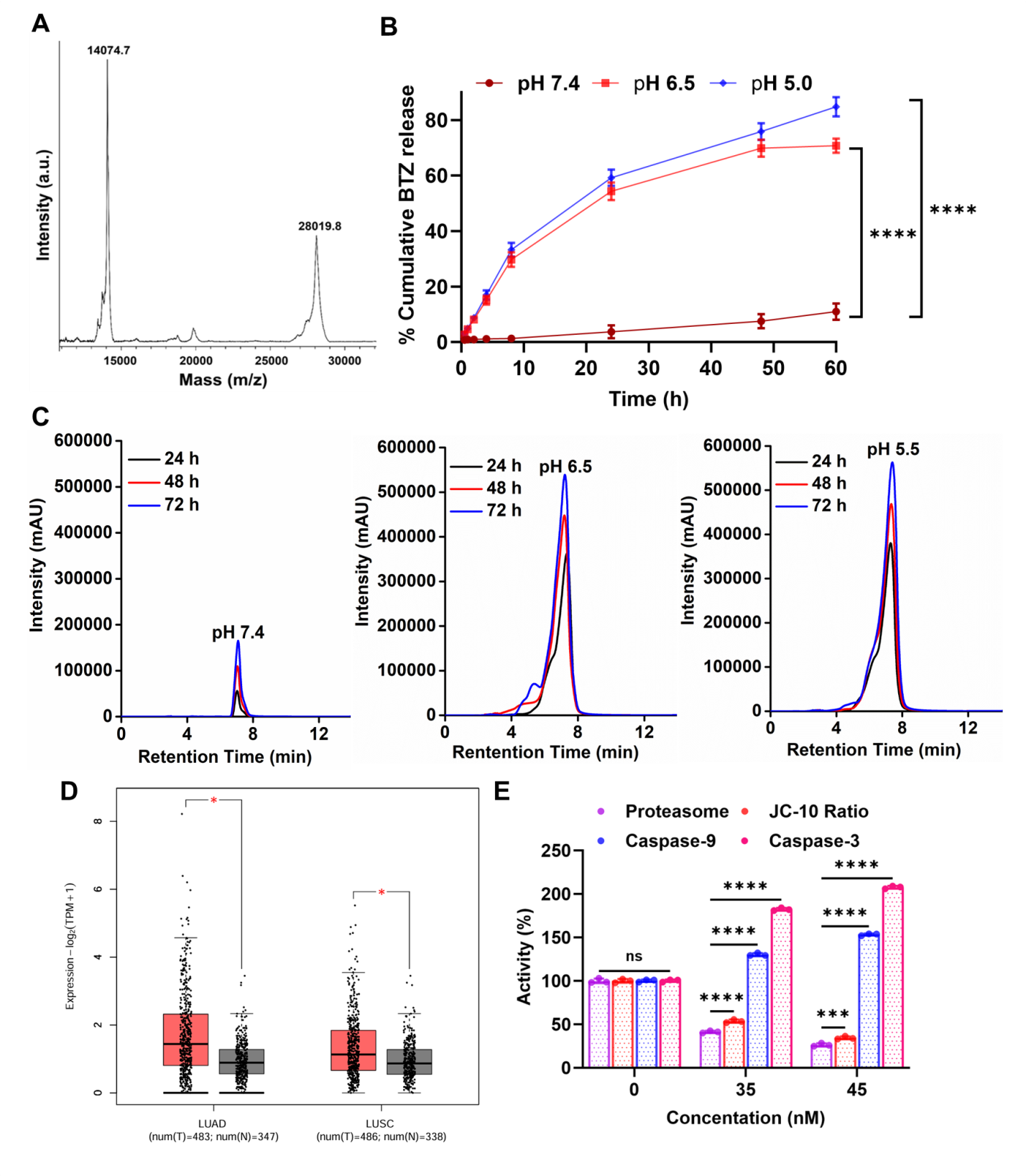
**

**Figure S6. Analytical characterization, ACE2 expression, BTZ release, and apoptotic cascade analysis.** A, Representative MALDI-TOF spectrum of the mRBD3dopa-BTZ conjugate. B, Cumulative BTZ release was measured using RP-HPLC at pH 7.4, 6.5, and 5.0. Data represent mean ± SD from three independent sample preparations, and each time point was measured in technical triplicate. Error bars denote standard deviation. Statistical analysis was made using a two-way ANOVA with Tukey’s multiple-comparison test showed significant differences among all pH conditions across the time course, ****p < 0.0001 are displayed for the final 60 h time point for clarity. C, HPLC chromatograms showing BTZ release from the mRBD3dopa-BTZ conjugate at 24, 48 and 60 h under pH 7.4, 6.5 and 5.0. D, Comparative expression of ACE2 in LUAD and LUSC versus normal lung tissue analyzed using GEPIA2 (TCGA + GTEx datasets). T represents tumor samples (red boxes) and N denotes normal/non-cancer samples (gray boxes); sample sizes are indicated. Both LUAD and LUSC tumors showed significantly higher ACE2 transcript levels than normal lung tissue (p < 0.05) for both comparisons. E, Mechanistic analysis of apoptosis: proteasome inhibition triggers mitochondrial damage and sequential caspase activation. The Data are presented as mean ± SD from at least three independent biological replicates, each done in four technical replicates. The data points representing the mean of each independent biological replicate are overlaid. Error bars denote standard deviation across the biological replicates. Statistical analysis was made using a two-way ANOVA with multiple comparisons and Tukey’s post hoc correction, ****p < 0.0001, ns = not significant.


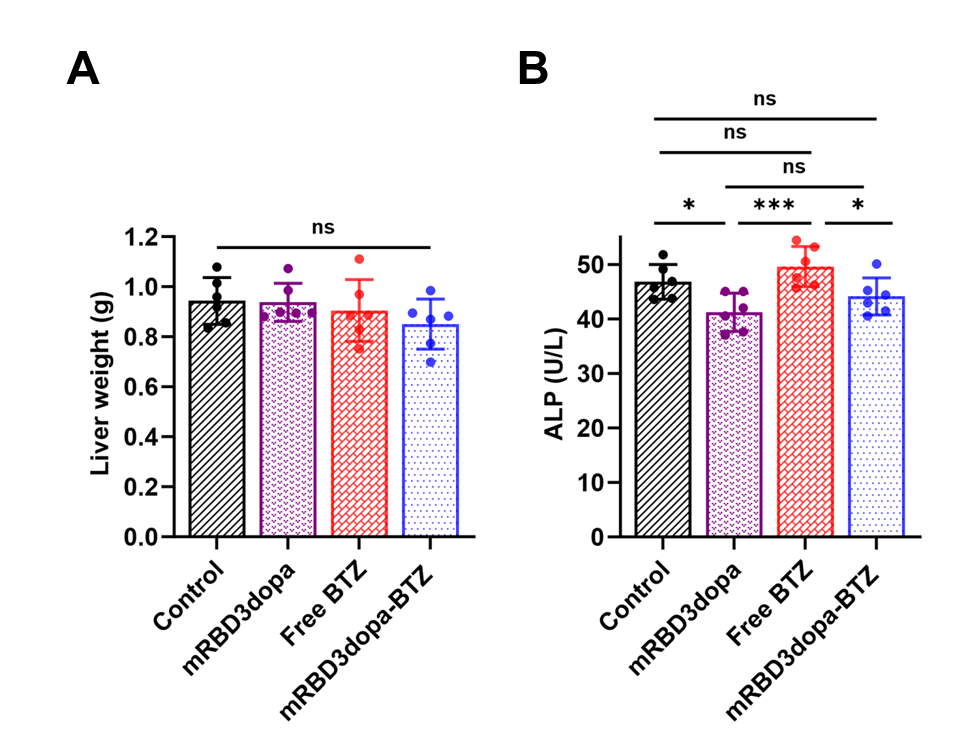


**Figure S7. Safety and selectivity of mRBD3dopa-BTZ conjugate *in vivo*.** A, Relative liver weight (g) of mice after 30 days of treatment. B, Evaluation of serum ALP levels. For all experiments, Data are presented as mean ± SD (biological replicates n = 6 mice per group). Statistical analysis was made using a one-way ANOVA with multiple comparisons and Tukey’s post hoc correction, *p< 0.05, ***p < 0.001, ns = not significant. Error bars represent standard deviation.

**Tables:**

**Table S1**. Rational design of RBD variants for site-specific delivery in cancer cells

| **Three distinct RBD variants** | **Amino acids positions in RBD** | **Amino acids in Wild type RBD** | **Amino acids in Mutant RBD** | **No. of mutations** | **Purpose / Rationale** | **Reference** |
| --- | --- | --- | --- | --- | --- | --- |
| Mutant RBD1 (mRBD1) | 326  374  460  478 | Ser | Thr | 4 | Increase protease resistance and conformational rigidity | (25, 104) |
| Mutant RBD2 (mRBD2) | 326  374  460  478 | Ser | Thr | 4 | Removed glycosylation sites to improve yield and reduce steric hindrance | (105, 106) |
|  | 332  344 | Asn | Ser | 1 |  |  |
|  |  |  | Thr | 1 |  |  |
| Mutant RBD3 (mRBD3) | 326  374  460  478 | Ser | Thr | 4 | Peripheral tyrosine substitutions were introduced to enable DOPA conjugation while maintaining structural stability and ACE2 binding affinity. | (107, 108) |
|  | 332  344 | Asn | Ser | 1 |  |  |
|  |  |  | Thr | 1 |  |  |
|  | 366  506 | Tyr | Trp | 2 |  |  |
|  | 422  474 |  | Phe | 2 |  |  |
|  | 490 |  | His | 1 |  |  |

**Table S2**. Docking score of RBDs and RBDdopa’s with ACE2

| **Proteins RBDs variants** | **Docking score of RBDs with ACE2 (kcal/mol)** | **Docking score of DOPA incorporated RBDs with ACE2 (kcal/mol)** |
| --- | --- | --- |
| nRBD | -787.7 | -787.7 |
| mRBD1 | -800.8 | -800.8 |
| mRBD2 | -862.3 | -862.3 |
| mRBD3 | -866.5 | -873.9 |

**Table S3.** The expected and observed molecular weight (MW) of RBD variants without modification

| **Proteins** | **Expected MW (Da)** | **Observed MW (Da)** |
| --- | --- | --- |
| nRBD | 25098.40 | 23686.559 |
| mRBD1 | 25154.51 | 24405.151 |
| mRBD2 | 25114.49 | 24112.785 |
| mRBD3 | 25102.53 | 24189.566 |
| ACE2 | 69769.82 | 69083.447 |

**Table S4.** Showing an amino acid modification in RBD variants, expected and observed molecular weight (MW) of RBD variants upon DOPA incorporation

| **DOPA incorporated RBDs** | **Modified amino acid (Tyr to DOPA)** | **Increase in MW (Da)** | **Expected MW (Da)** | **Observed MW (Da)** |
| --- | --- | --- | --- | --- |
| nRBDdopa | 15 | 15*16 =240 | 23929.559 | 23989.941 |
| mRBD1dopa | 15 | 15*16 =240 | 24645.151 | 24719.654 |
| mRBD2dopa | 15 | 15*16 =240 | 24352.785 | 24370.493 |
| mRBD3dopa | 10 | 10*16 =160 | 24349.566 | 24357.496 |

**Table S5.** MST analysis of both RBDs and DOPA-incorporated RBDs toward ACE2 is reflected in the apparent K_d_ values

| **Proteins RBDs interaction with ACE2** | **RBDs without genetic linker DOPA**  **k_D_ values (µM)** | **DOPA incorporated RBDs**  **k_D_ values (µM)** |
| --- | --- | --- |
| nRBD | 5.4 ± 0.4 | 1.8 ± 0.2 |
| mRBD1 | 3.9 ± 0.4 | 1.2 ± 5.8 |
| mRBD2 | 2.3 ± 1.3 | 7.1 ±0.8 |
| mRBD3 | 0.2 ± 0.5 | 0.9 ± 0.4 |

**Table S6.** Showing a molecular weight of mRBD3dopa-BTZ conjugate

|  | **MW (Da)** |
| --- | --- |
| mRBD3dopa | 24357.496 |
| BTZ | 3842.4 |
| Expected mRBD3dopa-BTZ | (24357.496 + 3842.4) - 180 = 28019.896 |
| Observed mRBD3dopa-BTZ | 28019.8 |

**Table S7.** Flow cytometric quantification of the apoptotic effect of conjugate and free BTZ in 2D cell culture. LL–Viable cells, LR-Early apoptotic cells, UR-Late Apoptotic cells and UL-Necrotic cells

| **Samples** | **A549 (Gated percentage)** | | | | **HSAEC (Gated percentage)** | | | |
| --- | --- | --- | --- | --- | --- | --- | --- | --- |
|  | **LL** | **LR** | **UL** | **UR** | **LL** | **LR** | **UL** | **UR** |
| Control | 99.69 | 0.00 | 0.31 | 0.00 | 100.00 | 0.00 | 0.00 | 0.00 |
| Free BTZ | 62.42 | 37.58 | 0.00 | 0.00 | 62.67 | 36.59 | 0.37 | 0.37 |
| mRBD3dopa-BTZ conjugate | 9.41 | 89.41 | 1.17 | 0.00 | 94.59 | 5.41 | 0.00 | 0.00 |

**Table S8.** Flow cytometric quantification of the apoptotic effect of conjugate and free BTZ in 3D A549 and HSAEC models. LL–Viable cells, LR-Early apoptotic cells, UR-Late Apoptotic cells and UL-Necrotic cells

| **Samples** | **3D A549 (Gated percentage)** | | | | **3D HSAEC (Gated percentage)** | | | |
| --- | --- | --- | --- | --- | --- | --- | --- | --- |
|  | **LL** | **LR** | **UL** | **UR** | **LL** | **LR** | **UL** | **UR** |
| Control | 95.16 | 4.84 | 0.00 | 0.00 | 98.54 | 0.00 | 1.46 | 0.00 |
| Free BTZ | 60.76 | 39.24 | 0.00 | 0.00 | 69.67 | 22.03 | 8.30 | 0.00 |
| mRBD3dopa-BTZ conjugate | 35.04 | 64.96 | 0.00 | 0.00 | 81.54 | 5.90 | 12.56 | 0.00 |
